# The association between social capital and self-care behaviors in patients with heart failure: A cross-sectional study

**DOI:** 10.1016/j.ijnsa.2025.100478

**Published:** 2025-12-21

**Authors:** Amirhossein Saem, Hamed Bazrafshan Drissi, Armin Sharifi, Javad Kojuri, Alireza Salehi

**Affiliations:** a Cardiovascular Research Center, School of Medicine, Shiraz University of Medical Sciences, Shiraz, Iran; b Associate Professor of Cardiology, Cardiovascular Research Center, School of Medicine, Shiraz University of Medical Sciences, Shiraz, Iran; c School of Medicine, Shiraz University of Medical Sciences, Shiraz, Iran; d Professor of Interventional Cardiology, Department of Cardiology, Clinical Education Research Center, Shiraz University of Medical Sciences, Shiraz, Iran; e Professor of Epidemiology, Department of MPH, School of Medicine, Shiraz University of Medical Sciences, Shiraz, Iran

**Keywords:** Heart failure, Social capital, Self-care, Behavioral medicine, Health psychology

## Abstract

**Background:**

Alongside pharmacological management, self-care is crucial for reducing morbidity and rehospitalization in heart failure patients. Previous studies have identified various medical, individual, and system barriers, particularly low social support. While social support focuses on the immediate patient environment (microsystem), social capital includes broader factors (exosystem, macrosystem), which remain unexplored in heart failure self-care.

**Methods:**

A multicenter cross-sectional study involving 157 heart failure patients (left ventricular ejection fraction≤50 or diastolic dysfunction) was conducted using stratified multistage sampling from hospitals and clinics affiliated with Shiraz University of Medical Sciences. Heart failure self-care was assessed using the Self-Care of Heart Failure Index (version 7.2), and social capital was measured via the 36-item Onyx and Bullen scale. Generalized linear models with leave-one-out cross-validation identified independent predictors of adequate self-care (score≥70), adjusting for demographic, clinical, and behavioral covariates, including self-care confidence.

**Results:**

Social capital scored relatively low to moderate (standardized median: 47.222, 95% confidence interval [95%CI]: 35.484–58.065). Higher social capital was positively correlated with self-care maintenance (*p* = 0.003), symptom perception (*p* = 0.019), and symptom management (*p* = 0.035). In multivariate models, community participation was associated with self-care maintenance (odds ratio [OR]: 1.026, 95%CI: 1.008–1.045, *p* = 0.004), symptom perception (OR: 1.025, 95%CI: 1.003–1.048, *p* = 0.028), and symptom management (OR: 1.025, 95%CI: 1.003–1.048, *p* = 0.024), all independent of confidence. Proactive engagement in social activities, also independent of confidence, was associated with symptom perception (OR: 1.031, 95%CI: 1.005–1.058, *p* = 0.021) and symptom management (OR: 1.038, 95%CI: 1.009–1.067, *p* = 0.009). This domain was associated with maintenance in models without confidence (OR: 1.027, 95%CI: 1.008–1.046, *p* = 0.005), but attenuated after adjustment for confidence, indicating the association was not independent of confidence. The significant association of community trust and safety with better maintenance and symptom perception also attenuated after accounting for confidence. Stronger work connections were also linked to these two domains, independent of confidence.

**Conclusions:**

Notably, most patients with heart failure exhibited inadequate self-care across all domains. Social proactivity and community participation, scarcely studied yet potentially modifiable aspects of social capital, were positively associated with self-care behaviors. From these findings, we suggest that integrating social capital assessments into multidisciplinary heart failure care can help nurses tailor behavioral interventions and strengthen community-based support strategies. Future interventional trials should evaluate targeted social-capital interventions as a novel adjunct to nursing-led self-care support in heart-failure patients.

**Registration:**

IR.SUMS.MED.REC.1404.205.

**What is already known about the topic**

• Higher social support and networks are linked with better self-care behaviors and confidence among patients with heart failure
• In other chronic conditions, higher levels of social capital (such as community participation, trust, and social networks) have been associated with improved self-care

Alt-text: Unlabelled box dummy alt text

**What this paper adds**

• We demonstrated the importance of social capital, including community participation, proactivity, community trust, and workplace relations, in the self-care of heart failure.
• We identified specific, modifiable social capital domains that were independently associated with different aspects of self-care of heart failure, even after adjusting for patient confidence and clinical factors.
• We found modifiable contributors to self-care of heart failure, providing a possible foundation for nurse-led interventions.

Alt-text: Unlabelled box dummy alt text

## 1 Introduction

Heart failure is a prevalent, chronic, and debilitating condition, defined as a multi-dimensional clinical syndrome encompassing signs and symptoms like exertional dyspnea, chronic fatigue, and body swelling primarily caused by structural or functional abnormalities in the cardiac capacity to be filled or pump blood effectively (Bragazzi et al., 2021; Heidenreich et al., 2022). As a non-communicable disease, heart failure continues to impose a significant global burden, characterized by high prevalence, mortality, and substantial healthcare costs associated with patient management (Bragazzi et al., 2021; Savarese et al., 2023). This syndromic condition mostly affects the elderly, who are more vulnerable due to coexisting comorbidities. These comorbidities, accompanying up to 30 % of heart failure patients, can inflict higher physical and mental burdens, medical expenses, morbidity, and mortality on patients as well as their family, friends, caregivers, surrounding people, healthcare system, and community (Chan et al., 2021; Savarese et al., 2023). Thus, it is imperative to adopt a multi-dimensional approach for the optimal management of heart failure.

The latest "2022 AHA/ACC/HFSA Guideline for the Management of Heart Failure" highlights the importance of multidisciplinary care, especially by nurses, social workers, dieticians, and primary care physicians (Heidenreich et al., 2022). These professionals can provide patient education and support, as well as interventions to address underlying factors contributing to poor self-care (Inam et al., 2025; Son et al., 2020). Proper self-care is crucial for patients with heart failure, as it directly impacts their quality of life, risk of hospitalization, and mortality rates (Jonkman et al., 2016; Ruppar et al., 2016). Researchers have identified factors such as multiple comorbidities, older age, male sex, lower income, lower education, and rural residence as person-related predictors of poor self-care (Niriayo et al., 2024; Zhang et al., 2023). Researchers also indicate that poor self-care habits are often linked to various social and psychosocial factors, such as isolation, depression, frailty, low health literacy, and inadequate social support and networks (D’Souza et al., 2025). These social factors can lead to unfavorable outcomes, particularly during the post-hospitalization transition phase (Heidenreich et al., 2022). Many experts suggest that social capital, which is a broader and more fundamental social determinant, may be responsible for these factors (Baptista et al., 2021; Mishra, 2020). Nurses can recognize these modifiable factors as part of discharge planning and outpatient follow-up, providing targeted support, motivation, education, and linkage to community resources. Huang et al. (2022) found in a meta-analysis that nurse-led interventions can improve self-care behaviors among heart failure patients.

According to the Situation-Specific Theory of Heart Failure Self-care, self-care is a “naturalistic decision-making” process that facilitates three linked behaviors among heart failure patients (Riegel and Dickson, 2008). These behaviors include (I) self-care maintenance, maintaining physiological stability, (II) symptom perception, monitoring and interpreting changes, and (III) self-care management, responding to symptoms when they occur. Confidence (self-efficacy) plays a moderating or mediating role in these processes, and patients’ characteristics, disease nature, and environmental factors influence decision-making and self-care actions (Riegel et al., 2022, 2016; Riegel and Dickson, 2008). This theoretical framework provided a useful lens for our study: we consider self-care behaviors as key outcomes and conceptualize social capital as an environmental/contextual factor that may influence those outcomes, alongside known demographic and behavioral covariates.

Social capital is defined as a “bottom-up” phenomenon resulting from everyday interactions between people that builds social networks, trust, and norms (Onyx and Bullen, 2000). It is a concept different from social support. Social capital is often conceptualized as a dynamic resource that resides within social networks and is gained through trustworthy relationships, kind acts, and social collaboration (Reyes et al., 2020). Having reciprocal ties, the social capital is prompted by the exchange of information and social support within a community. Put simply, social capital defines people’s capacity to bond together within their social networks, receive or offer support, and bridge new links to unfamiliar organizations like professional multidisciplinary caregiving support teams, nursing homes, cardiac rehabilitation, online health communities, and other medical care providers (Baptista et al., 2021; Bryant, 2012; Loane and D’Alessandro, 2013).

Prior researchers have emphasized the positive impact of social capital on self-care of various chronic illnesses, such as diabetes, hypertension, chronic angina, and chronic lung disease (Christian et al., 2020; Waverijn et al., 2017). Social capital may help patients to deal with their chronic illnesses within a network of mutual trust, shared norms, and cooperative relationships (Amoah and Oteng, 2024; Onyx and Bullen, 2000). For instance, a realist review found that social networks and social capital played an important role in self-management of chronic illnesses (Vassilev et al., 2011). Another group of researchers revealed that neighborhood social capital was positively associated with improved self-management of chronic illnesses and may mediate the relationship between social capital and health outcomes (Waverijn et al., 2017). More broadly, researchers conducting systematic reviews have supported that higher social capital correlates with better physical and mental health and reduced mortality.

Despite this growing evidence in other chronic diseases, the existing literature lacks direct investigation of social capital in relation to self-care among patients with heart failure (Ehsan et al., 2019). Additionally, previous researchers have primarily examined social networks and social support among this patient group, whereas social capital represents a broader construct encompassing these social determinants (Babygeetha and Devineni, 2024; Kleman, 2024). Given the limited research on social capital and its domains in the self-care of heart failure patients, we aimed to address this gap by using validated instruments that capture community participation, trust, safety, and civic/work engagement (Bryant, 2012; Heidenreich et al., 2022).

Guided by the Situation-Specific Theory of Heart Failure Self-Care, we hypothesized the following: **Hypothesis 1:** Higher social capital is associated with better self-care among patients with heart failure, independent of demographic, clinical, and behavioral covariates. **Hypothesis 2:** Specific dimensions of social capital are associated with better self-care of heart failure, independent of demographic, clinical, and behavioral covariates. We also investigated whether the association between social capital and self-care behaviors remained independent of self-care confidence, illustrating the theoretical function of confidence in the self-care decision-making process.

## 2 Materials and methods

### 2.1 Study design

The present study was a multicenter, community-based, and cross-sectional investigation aimed at exploring the correlation between social capital and the self-care of heart failure index, considering their related dimensions. This study is noteworthy for including both inpatient and outpatient participants, in contrast to the majority of prior studies that solely focused on hospitalized patients or a single subset of heart failure patients (Graven et al., 2015). Furthermore, incorporating multiple noninstitutional private clinics, in addition to public clinics, allowed us to capture patients living in the community. Therefore, we consider the study community-based, as it reflects a broader spectrum of heart failure patients beyond those currently hospitalized.

### 2.2 Participants and setting of patients

The study population consisted of patients with chronic heart failure who visited specialized cardiovascular clinics, both public clinics affiliated with Shiraz University of Medical Sciences and private clinics, for routine follow-up, as well as those admitted to the coronary care units of Shiraz University hospitals due to acute decompensated heart failure. The inclusion criteria were as follows: adult patients hospitalized due to primary or secondary causes of heart failure, in addition to non-hospitalized patients, confirmed by a cardiologist's report based on echocardiographic findings indicating reduced left ventricular ejection fraction (≤ 40 %), mildly reduced ejection fraction (between 40 % and 50 %) or abnormal diastolic dysfunction (ejection fraction ≥ 50 %), as well as those under standard pharmacological treatment for heart failure for at least 2 months. Left ventricular ejection fraction was quantified employing the biplane Simpson’s method. Diastolic function assessment was conducted through analysis of mitral inflow velocities (the ratio of peak early diastolic filling velocity to the peak velocity produced by atrial contraction, E/A ratio), pulmonary venous flow, and tissue Doppler imaging. Additionally, participants were required to be Iranian nationals, fluent in Persian, and capable of understanding and responding to questions in the Persian language. Exclusion criteria included recent acute coronary syndrome within the past 40 days, as diagnosed by the research physician, and conditions that might impair cognitive ability or communication, such as dementia, intellectual disability, stroke, severe psychiatric disorders, or any neurological condition leading to significant impairment in consciousness or comprehension.

We employed a stratified multistage cluster sampling approach to address a key limitation of previous research that had focused exclusively on either hospitalized patients or outpatient clinic populations (Graven et al., 2015). Our design intentionally incorporated both groups. In the first stage, patients were stratified via proportionate sampling into two primary strata: (1) hospitalized patients and (2) outpatient department patients. During the second stage, hospitalized patients were further subdivided into two hospital-based strata: (I) coronary care units of Shiraz Shahid-Faghihi Hospital, and (II) coronary care units of Shiraz Namazi Hospital, both located in Fars, Iran. To determine proportional representation, we conducted randomized audits over 3 distinct days across 3 consecutive months to estimate admission rates in each center prior to patient recruitment. For outpatient sampling, we established two clinic-based strata to control for socioeconomic bias and ensure coverage of both high and low socioeconomic groups: (I) patients from the public Imam Reza Specialty and Subspecialty Clinic in Shiraz and (II) patients from private cardiology clinics. In the third stage, we compiled master lists of practicing cardiologists from both settings (public and private) and randomly selected two specialists from each stratum using simple random sampling. Finally, quota sampling was applied to recruit patients from each of the four selected physicians' practices to complete our study population. Quota sampling was applied to ensure that the sample size within each stratum matched the proportion found in the general population, and simple random sampling was used for the final selection of participants. This methodological approach facilitated a comprehensive analysis of the variables under study, ensuring that the results were representative of the target population. Importantly, both symptomatic and asymptomatic patients were included in the study, encompassing heart failure patients with stage B (pre-heart failure), stage C (symptomatic heart failure), and stage D (advanced heart failure).

### 2.3 Sampling size

Since the study’s primary aim was to obtain the correlation coefficient between the two variables, social capital and self-care, the overall sample size was determined using the following formula:

(1) $$N=\;\frac{{\left(z_{1-\alpha }\;+\;z_{1-\beta }\right)}^{2}}{u_{\rho }^{2}}+3$$

(2) $$u_{\rho }=\;\frac{1}{2}\log\frac{1+\rho }{1-\rho }+\frac{\rho }{2\;\left(N-1\right)}$$

(3) $$u_{\rho }^{0}=\;\frac{1}{2}\log\left[\frac{1+\rho }{1-\rho }\right]$$

The required sample size was initially calculated to be 84 participants based on a correlation coefficient (ρ) of 0.3, considering moderate correlation (Cohen, 1988), a significance level (α) of 0.05, and a desired statistical power (1-ß) of 80 %. Accounting for the six covariates included in the analysis and applying the standard criterion of 10 additional samples per covariate, the final adjusted sample size was determined to be at least 144 heart failure patients. To ensure an adequate sample size for comparing the mean self-care behaviors between two groups of heart failure patients (adequate *versus* inadequate) while accounting for potential covariates, we calculated the required sample size using an independent two-sample *t*-test framework. The formula applied was:

(4) $$m=\;\left(\frac{1+\varphi }{\varphi }\right)\frac{{\left(z_{1-\frac{\alpha }{2}}+z_{1-\beta }\right)}^{2}}{\Delta^{2}}+\frac{z_{1-\frac{\alpha }{2}}^{2}}{2\left(1+\varphi \right)}$$

The required sample size for the second group (*n*) was defined by this formula: n=φm. Hence, the total sample size was calculated this way: N=m+φm. The allocation ratio (φ) was set at 3.167, based on previous studies (Schäfer-Keller et al., 2021), and the effect size was regarded as 0.5, considering a moderate effect size (Cohen, 1988). Following the application of Eq. (4), the least required sample size was calculated to be 150 heart failure patients.

### 2.4 Data measuring instruments

A group of trained researchers conducted comprehensive interviews with all participating patients at both clinics and hospitals. To ensure consistency, all interviewers received training on the administration of both questionnaires, interview techniques, and scoring algorithms before study initiation. Following training, the team completed a pilot phase with 10 patients to align interviewing styles, ensure that questions were clearly understood, and implement appropriate operational procedures. During data collection, the principal investigator closely supervised the team, reviewing completed questionnaires weekly to check for completeness, logical consistency, and scoring accuracy. Any discrepancies or uncertainties were addressed in consensus meetings, and feedback was provided to correct and improve the process. These measures helped ensure that data collection was uniform and of high quality across all study sites.

During interviews, demographic variables such as age, sex, marital status, perceived income level, residential area, educational level, and duration of cardiologist surveillance were collected. Additionally, clinical parameters encompassing heart failure subtype and ejection fraction were extracted from the most recent transthoracic echocardiography performed within 6 months prior to interviews. The medical history of participants was meticulously documented during interviews, noting the presence of conditions such as diabetes, hypertension, hyperlipidemia, chronic renal failure, chronic obstructive pulmonary disease, and thyroid disorders. Furthermore, social history factors, including tobacco use, waterpipe smoking, and opioid consumption, were also questioned. Participants were also interviewed using two standardized questionnaires designed to measure self-care of heart failure and social capital, with patient interviews occurring from June 2025 to August 2025.

#### 2.4.1 Self-Care of heart failure index

Among the most widely used instruments for measuring self-care in heart failure patients is the Self-Care of Heart Failure Index (Vellone et al., 2020), with its latest version (version 7.2) translated into 22 languages, including Persian. This 39-item questionnaire assesses three domains: maintenance behaviors (10 items: actions taken to maintain health and prevent deterioration), management behaviors (8 items: strategies for handling symptoms and managing health crises), and symptom perception (11 items: patients’ awareness and interpretation of their symptoms), plus 10 items evaluating self-care confidence (evaluates the confidence patients feel in their ability to care for themselves). Respondents evaluate each item utilizing a 5-point Likert scale ranging from 1 (never) to 5 (always). As per the official algorithm proposed in version 7.2, standardized scoring involves summing raw scores per domain, subtracting the minimum possible score, dividing by the score range, and then multiplying by 100. Using a 70-point cutoff, patients are classified as having adequate (≥70) or inadequate (<70) self-care in each domain. In line with the official scoring instructions, a total score was not computed for the self-care of heart failure index, as the three behavioral domains are conceptually distinct. Furthermore, in accordance with the conceptual model proposed by Vellone et al. (2020), the self-care confidence domain was analyzed separately as an influencing factor rather than included in the self-care behavior scores. The Persian version was obtained after completing the Instrument Use Agreement from self-care-measures.com.

#### 2.4.2 Onyx and Bullen social capital scale

To assess social capital, the Onyx and Bullen (2000) questionnaire was employed. This 36-item instrument evaluates eight dimensions, with respondents rating each item on a 4-point Likert scale ranging from 1 (very low) to 4 (very high); including: Community Participation (7 items); Pro-Activity in Social Context (7 items); Feelings of Trust and Safety (5 items); Neighborhood Connections (5 items); Family and Friends Connections (3 items); Tolerance of Diversity (2 items); Value of Life (2 items); and Work Connections (3 items). Two additional items contribute to the total social capital score without belonging to any specific dimension. Notably, items 32–36 apply only to employed individuals and are excluded for unemployed or retired respondents. Standardized scores for each dimension are calculated by summing raw scores, subtracting the minimum possible score, dividing by the possible score range, and multiplying by 100. The total social capital score follows the same calculation method. The Persian version's reliability and validity were confirmed by Eftekharian et al. (2016) for the elderly population.

### 2.5 Statistical methods

Statistical analyses were performed using IBM SPSS Statistics, version 27.0 and R Statistical Software, version 4.4.3. Descriptive statistics, including median, interquartile range (IQR), frequency, and percentage, were calculated for all variables. The Pearson correlation coefficient test was used to examine the relationship between social capital and self-care behaviors. For non-normally distributed data, the Kendall Rank correlation coefficient test was employed. To compare self-care behaviors between the adequate and inadequate groups (based on the 70-point cutoff), the independent two-sample *t*-test was used for normally distributed data, and the Mann-Whitney U test was applied for non-normally distributed data. In all analyses, a *p-*value <0.05 was considered statistically significant.

Missing data were addressed using bagged tree imputation from the “tidymodels” package, version 1.3.0 (Kuhn and Wickham, 2020) within R Statistical Software. For the multivariate analysis, generalized linear models with a binomial distribution (adequate *versus* inadequate self-care) and a logit link function were employed through the Classification and Regression Training (caret) package, version 7.0–1 (Kuhn and Max, 2008). This was combined with the leave-one-out cross-validation method for internal validation, and the optimal model was selected based on the highest area under the receiver operating characteristic curve. The leave-one-out cross-validation technique maximizes data utilization by iteratively training the model on N-1 observations and validating on the one left out. This method helps reduce variance in performance estimates while avoiding the data loss inherent in traditional train-test splits or k-fold cross-validation. For variable selection, all potential confounders, including self-care confidence, care setting (hospitals or clinics), sex, age, marital status, residential location (rural or urban), income level, educational level, heart failure subtype, addictions, left ventricular ejection fraction, duration of cardiologist surveillance, and number of comorbidities, were initially incorporated in model training. Then, step-wise backward elimination was performed based on the Wald test *p-*value, implemented using the “blorr” package. Variables with *p-*values above 0.25 were iteratively removed until all remaining variables met the retention criterion (Hebbali, 2024). The collinearity assumption was assessed by calculating the variance inflation factor (VIF) for each predictor, ensuring all predictors had VIF values below 5. In cases where the VIF exceeded this threshold, highly correlated predictors (correlation exceeding 0.8) were identified via the correlation matrix, appropriately combined, and subsequently reassessed. For data visualization, the “ggplot2” package, version 3.5.2 (Wickham, 2016) and the “ggstatsplot” package, version 0.13.1 (Patil, 2021) in RStudio, version 2024.12.0 + 467, were utilized to create the included bar charts and box plots.

### 2.6 Ethical considerations

The study protocol received approval from the ethics committees of all participating hospitals and clinics, as well as from the Research Ethics Committees of Shiraz University of Medical Sciences – School of Medicine, which approved the present study under the following number: IR.SUMS.MED.REC.1404.205. Furthermore, informed consent was obtained from every participant enrolled during the questionnaire interview.

## 3 Results

A total of 157 participants were enrolled in the study, comprising 89.17 % (*n* = 140) symptomatic and 10.83 % (*n* = 17) asymptomatic heart failure patients. Heart failure with reduced ejection fraction was more prevalent than heart failure with mildly reduced ejection fraction and heart failure with preserved ejection fraction. The median ejection fraction values were observed to be 28.08 % (IQR: 20 %–35 %) for heart failure with reduced ejection fraction, 45 % (IQR: 43.12 %–45 %) for heart failure with mildly reduced ejection fraction, and 57.5 % (IQR: 50 %–61.25 %) for heart failure with preserved ejection fraction. There was no significant difference between heart failure patients with reduced, mildly reduced, or preserved ejection fractions regarding the adequacy of self-care domains, as detailed in Table 1 (all *p-*values>0.05). Age, sex, educational level, and perceived income did not significantly influence the adequacy of self-care domains (all *p-*values>0.05; see Table 1). Hypertension was the most common comorbidity among heart failure patients, affecting 44.59 % (*n* = 70), followed by diabetes, which affected 36.31 % (*n* = 57). Additionally, having comorbidities, such as hypertension, diabetes, hyperlipidemia, chronic renal failure, and thyroid diseases, did not impact sufficiency of self-care domains, as detailed in Table 1 (all *p-*values>0.05). However, heart failure patients who suffered from chronic obstructive pulmonary disease exhibited a higher risk of inadequate self-care confidence, with an odds ratio (OR) of 10.17 (95 % CI: 1.38–450.97) and a chi-square *p-*value of 0.008.

**Table 1:** Demographic, Social, Medical, and Clinical Characteristics of Heart Failure Patients, Stratified by the Adequacy of Self-Care Domains and Self-Care Confidence.

Patients Characteristics		Total (*N* = 157)	Maintenance Scale of SCHFI		Symptom Perception Scale of SCHFI		Symptom Management Scale of SCHFI		Self-Care Confidence	
			Inadequate (*N* = 93)	Adequate (*N* = 64)	Inadequate (*N* = 107)	Adequate (*N* = 33)	Inadequate (*N* = 100)	Adequate (*N* = 40)	Inadequate (*N* = 82)	Adequate (*N* = 75)
Age, median (IQR)		64 (56– 71)	64 (57–70)	63.5 (55.75–72)	64 (56–70)	64 (58–72)	64 (56–71)	64.93 (58–72)	65.5 (56–71)	63 (57.5–71)
	*p-*values*	–	0.969^1^		0.648^1^		0.472^1^		0.436^1^	
Sex										
	Male, *n* ( %)	104 (66.24 %)	60 (38.22 %)	44 (28.03 %)	69 (49.29 %)	20 (14.29 %)	65 (46.43 %)	24 (17.14 %)	53 (33.76 %)	51 (32.48 %)
	Female, *n* ( %)	53 (33.76 %)	33 (21.02 %)	20 (12.74 %)	38 (27.14 %)	13 (9.29 %)	35 (25 %)	16 (11.43 %)	29 (18.47 %)	24 (15.29 %)
	*p-*values	–	0.704^2^		0.843^2^		0.718^2^		0.782^2^	
Residential Area										
	Urban Areas, *n* ( %)	129 (82.17 %)	70 (44.59 %)	59 (37.58 %)	85 (60.71 %)	28 (20 %)	80 (57.14 %)	33 (23.57 %)	69 (43.95 %)	60 (38.22 %)
	Rural Area (Villages and Tribes), *n* ( %)	28 (17.83 %)	23 (14.65 %)	5 (3.18 %)	22 (15.71 %)	5 (3.57 %)	20 (14.29 %)	7 (5 %)	13 (8.28 %)	15 (9.55 %)
	*p-*values	–	0.012^2^		0.663^2^		0.919^2^		0.639^2^	
Setting										
	Public OPD, *n* ( %)	43 (27.39 %)	28 (17.83 %)	15 (9.55 %)	29 (20.71 %)	5 (3.57 %)	25 (17.86 %)	9 (6.43 %)	22 (14.01 %)	21 (13.78 %)
	Private OPD, *n* ( %)	99 (63.06 %)	55 (35.03 %)	44 (28.03 %)	68 (48.57 %)	24 (17.14 %)	67 (47.86 %)	25 (17.86 %)	50 (31.85 %)	49 (31.21 %)
	Hospitalized, *n* ( %)	15 (9.55 %)	10 (6.37 %)	5 (3.18 %)	10 (7.14 %)	4 (2.86 %)	8 (5.71 %)	6 (4.29 %)	10 (6.37 %)	5 (3.18 %)
	*p-*values	–	0.469^2^		0.368^2^		0.458^2^		0.499^2^	
Education Level										
	Illiterate, *n* ( %)	37 (23.57 %)	21 (13.38 %)	16 (10.19 %)	27 (19.29 %)	9 (6.43 %)	22 (15.71 %)	14 (10 %)	22 (14.01 %)	15 (9.55 %)
	Primary School, *n* ( %)	74 (47.13 %)	47 (29.94 %)	27 (17.20 %)	52 (37.14 %)	15 (10.71 %)	50 (35.71 %)	17 (12.14 %)	40 (25.48 %)	34 (21.66 %)
	Diploma, *n* ( %)	26 (16.56 %)	15 (9.55 %)	11 (7.01 %)	15 (10.71 %)	4 (2.86 %)	12 (8.57 %)	7 (5 %)	9 (5.73 %)	17 (10.83 %)
	Higher than Diploma, *n* ( %)	20 (12.74 %)	10 (6.37 %)	10 (6.37 %)	13 (9.29 %)	5 (3.57 %)	16 (11.43 %)	2 (1.43 %)	11 (7.01 %)	9 (5.73 %)
	*p-*values	–	0.709^2^		0.953^2^		0.136^2^		0.244^2^	
Perceived Level of Income										
	Lower than Average, *n* ( %)	100 (63.69 %)	61 (38.85 %)	39 (24.84 %)	72 (51.43 %)	23 (16.43 %)	66 (47.14 %)	29 (20.71 %)	57 (36.31 %)	43 (27.39 %)
	Average, *n* ( %)	53 (33.76 %)	31 (19.75 %)	22 (14.01 %)	31 (22.14 %)	10 (7.14 %)	31 (22.14 %)	10 (7.14 %)	23 (14.65 %)	30 (19.11 %)
	Higher than Average, *n* ( %)	4 (2.55 %)	1 (0.64 %)	3 (1.91 %)	4 (2.86 %)	0 (0 %)	3 (2.14 %)	1 (0.71 %)	2 (1.27 %)	2 (1.27 %)
	*p-*values	–	0.353^2^		0.530^2^		0.758^2^		0.276^2^	
Social History										
	Smoking, *n* ( %)	46 (29.30 %)	28 (17.83 %)	18 (11.46 %)	32 (22.86 %)	8 (5.71 %)	31 (22.14 %)	9 (6.43 %)	21 (13.38 %)	25 (15.92 %)
	*p-*values	–	0.789^2^		0.529^2^		0.315^2^		0.282^2^	
	Waterpipe, *n* ( %)	14 (8.92 %)	11 (7.01 %)	3 (1.91 %)	9 (6.43 %)	4 (2.86 %)	11 (7.86 %)	2 (1.43 %)	7 (4.46 %)	7 (4.46 %)
	*p-*values	–	0.123^2^		0.521^2^		0.269^2^		0.861^2^	
	Opioids, *n* ( %)	19 (12.10 %)	11 (7.01 %)	8 (5.10 %)	15 (10.71 %)	3 (2.14 %)	12 (8.57 %)	6 (4.29 %)	8 (5.10 %)	11 (7.01 %)
	*p-*values	–	0.899^2^		0.460^2^		0.632^2^		0.346^2^	
Comorbidities										
	Diabetes, *n* ( %)	57 (36.31 %)	34 (21.66 %)	23 (14.65 %)	39 (27.86 %)	16 (11.43 %)	37 (26.43 %)	18 (12.86 %)	34 (21.66 %)	23 (14.65 %)
	*p-*values	–	0.937^2^		0.216^2^		0.381^2^		0.160^2^	
	Hypertension, *n* ( %)	70 (44.59 %)	41 (26.11 %)	29 (18.47 %)	42 (30 %)	19 (13.57 %)	44 (31.43 %)	17 (12.14 %)	36 (22.93 %)	34 (21.66 %)
	*p-*values	–	0.879^2^		0.063^2^		0.872^2^		0.875^2^	
	Hyperlipidemia, *n* ( %)	47 (29.94 %)	30 (19.11 %)	17 (10.83 %)	33 (23.57 %)	12 (8.57 %)	31 (22.14 %)	14 (10 %)	25 (15.92 %)	22 (14.01 %)
	*p-*values	–	0.444^2^		0.553^2^		0.647^2^		0.875^2^	
	Chronic Renal Failure, *n* ( %)	23 (14.65 %)	11 (7.01 %)	12 (7.64 %)	15 (10.71 %)	6 (4.29 %)	16 (11.43 %)	5 (3.57 %)	11 (7.01 %)	12 (7.64 %)
	*p-*values	–	0.228^2^		0.558^2^		0.600^2^		0.647^2^	
	COPD, *n* ( %)	11 (7.01)	8 (5.10 %)	3 (1.91 %)	9 (6.43 %)	2 (1.43 %)	9 (6.43 %)	2 (1.43 %)	10 (6.37 %)	1 (0.64 %)
	*p-*values	–	0.345^2^		0.661^2^		0.427^2^		0.008^2^	
	Thyroid Diseases, *n* ( %)	18 (11.46 %)	11 (7.01 %)	7 (4.46 %)	11 (7.86 %)	6 (4.29 %)	12 (8.57 %)	5 (3.57 %)	9 (5.73 %)	9 (5.73 %)
	*p-*values	–	0.863^2^		0.224^2^		0.935^2^		0.841^2^	
	Number of Comorbidities, median [IQR]	2 [1–3]	2 [1–3]	2 [1–3]	2 [1–3]	3 [2 – 4]	2 [1–4]	2 [2 – 3]	2 [1–3]	2 [1–4]
	*p-*values	–	0.845^1^		0.066^1^		0.643^1^		0.436^1^	
Subtype of Heart Failure										
	Systolic Dysfunction (HFrEF), *n* ( %)	135 (85.99 %)	79 (50.32 %)	56 (35.67 %)	91 (65 %)	30 (21.43 %)	84 (60 %)	37 (26.43 %)	68 (43.31 %)	67 (42.68 %)
	HFmrEF, *n* ( %)	14 (8.92 %)	10 (6.37 %)	4 (2.55 %)	12 (8.57 %)	0 (0 %)	11 (7.86 %)	1 (0.71 %)	10 (6.37 %)	4 (2.55 %)
	Diastolic Dysfunction (HFpEF), *n* ( %)	8 (5.10 %)	4 (2.55 %)	4 (2.55 %)	4 (2.86 %)	3 (2.14 %)	5 (3.57 %)	2 (1.43 %)	4 (2.55 %)	4 (2.55 %)
	*p-*values	–	0.556^2^		0.073^2^		0.266^2^		0.321^2^	
Left Ventricular Ejection Fracture, median (IQR)		30 (23.39– 40)	33 (20 – 40)	29.09 (25 – 35)	30 (20 – 40)	25 (20 – 35)	30 (20 –39.25)	26.37 (20 – 35)	31 (24.25 – 40)	27.9 (23.3 – 35)
	*p-*values	–	0.738^1^		0.269^1^		0.397^1^		0.146^1^	
Number of Years being under Surveillance of Cardiologist, median (IQR)		6 (2 – 11)	6 (2 – 11)	5.5 (2 – 12.125)	5 (2 – 11)	9 (5 – 15)	6.13 (2 – 11)	7.5 (3.75 – 15)	6 (2 – 11.75)	5 (2 – 10.99)
	*p-*values	–	0.748^1^		0.010^1^		0.178^1^		0.947^1^	

⁎ All p-values less than 0.05 are considered statistically significant.

1 P-values of the Kruskal-Wallis test, a non-parametric test, compares mean ranks between inadequate and adequate subgroups.

2 P-values of the Chi-squared tests. Abbreviations. SCHFI: self-care of heart failure index, COPD: chronic obstructive pulmonary disease, HFrEF: heart failure with reduced ejection fraction, HFpEF: heart failure with preserved ejection fraction, HFmrEF: heart failure with mildly reduced ejection fraction.

The majority of heart failure patients demonstrated inadequacy in three domains of self-care, including self-care maintenance (59.24 %), symptom perception (76.43 %), and symptom management (71.43 %), as well as in self-care confidence (52.23 %). As demonstrated in **Supplementary Material,** *Figure S1*, the residential area influenced self-care maintenance of heart failure patients. Notably, individuals residing in villages and tribal areas had lower self-care maintenance, with a median of 58.75 (IQR: 42.5–67.5), compared to those living in cities, with a median of 67.5 (IQR: 52.5–77.5). The left ventricular ejection fraction did not significantly impact the adequacy of self-care of heart failure patients across its domains, as detailed in Table 1 (all Kruskal-Wallis *p-*values>0.05). Notably, heart failure patients with adequate symptom perception scores had been monitored by their cardiologist for a longer duration, as depicted in **Supplementary Material,** *Figure S2*. Subgroup comparisons of self-care and social capital scores by care setting (hospital or clinic) are presented in **Supplementary Material,** *Table S1*. No significant differences were observed in any of the self-care domains or in self-care confidence between patients recruited from hospitals and clinics. Hence, the sample was considered sufficiently homogeneous for subsequent analyses, with care setting included as a covariate to account for any potential residual effects. Regarding social capital, the sole different domain of social capital was feeling of safety and trust, which was lower among hospitalized patients (53.33 [33.33–60] *versus* 60 [46.67–80], *p* = 0.008).

### 3.1 Role of social capital in self-care of heart failure

**Fig. 1:**
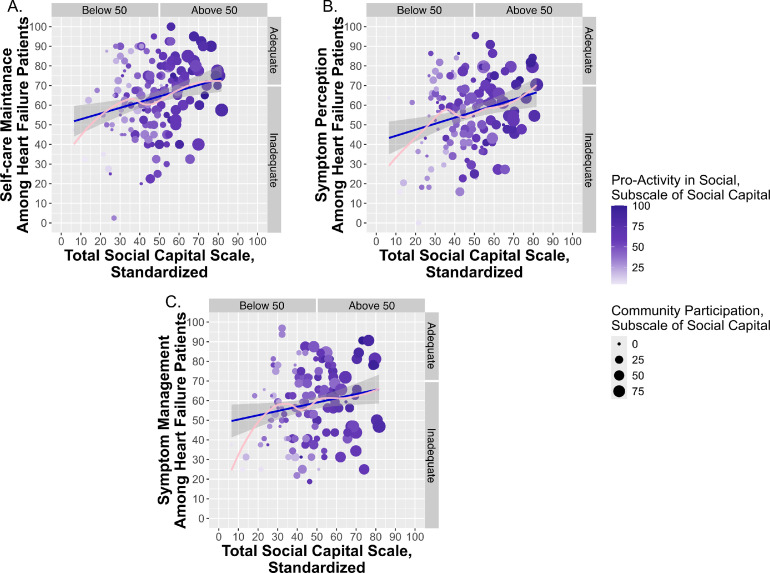
Relationship between social capital and self-care in patients with heart failure. **(A)** Scatterplot showing the association between total social capital (standardized score) and self-care maintenance behaviors (standardized score). **(B)** Scatterplot showing the association between total social capital (standardized score) and symptom perception domain of self-care of heart failure (standardized score). **(C)** Scatterplot showing the association between total social capital (standardized score) and symptom management domain of self-care of heart failure (standardized score). Point color represents the *Pro-Activity in Social* subscale of social capital (darker shades indicate higher scores), and point size reflects the *Community Participation* subscale of social capital (larger circles indicate higher scores). The fitted regression lines are shown in blue and pink, with shaded areas indicating 95 % confidence intervals.

Among the evaluated heart failure patients, overall social capital was relatively low to moderate, with a standardized median score of 47.222 (95 % CI: 35.484–58.065). The results of collinearity tests revealed a positive linear relationship between scores of total social capital and all three domains of self-care. This correlation is visually depicted in Fig. 1**A–C**, which demonstrates that as social capital increased among heart-failure patients, their self-care behaviors concomitantly increased. The total score of social capital was significantly correlated with maintenance behaviors (Spearman’s rho=0.233, 95 % CI: 0.079–0.376, *p* = 0.003), symptom perception (Pearson’s *r* = 0.260, 95 % CI: 0.098–0.408, *p* = 0.002), and symptom management (Pearson’s *r* = 0.183, 95 % CI: 0.018–0.339, *p* = 0.030). As illustrated in Fig. 1A, heart failure patients demonstrating higher maintenance scores also exhibit greater proactive engagement in social activities (represented by darker circles) and increased community participation (represented by larger circles). Fig. 1B and 1C further elucidate the positive correlation of social capital with both symptom perception and symptom management.

Notably, heart failure patients with adequate maintenance behaviors reported higher total social capital, with a median of 54.20 (IQR: 42.85–63.35) compared to their counterparts with inadequate maintenance, with a median of 44.09 (IQR: 32.26–51.86) (*p* < 0.001). Similarly, patients with adequate symptom perception had a median total social capital score of 54.63 (IQR: 38.89–60.22), while those with inadequate perception scored lower, with a median of 44.44 (IQR: 34.95–54.30) (*p* = 0.036). Higher total social capital scores were also observed in patients with adequate symptom management (median: 50.73, IQR: 40.83–58.06) compared to those with inadequate management (median: 44.44, IQR: 34.37–55.65), reaching statistical significance in multivariate analysis, when accounting for potential confounding variables (adjusted *p* = 0.004). Furthermore, self-care confidence was significantly correlated with total social capital scores (Spearman’s rho=0.329, 95 % CI: 0.175–0.446, *p* < 0.001). Given its role as a mediator in self-care behaviors, self-care confidence was included in subsequent analyses to assess its contribution to the associations observed between social capital and the three self-care domains.

Multivariate analysis further validated the initial findings regarding the associations between social capital and self-care behaviors across maintenance, symptom perception, and symptom management domains. After adjusting for potential covariates, including age, sex, residential area, care setting, education level, income level, addictions, comorbidities, heart failure subtype, ejection fraction, and years of cardiologist surveillance, the total social capital score remained significantly associated with higher odds of adequate self-care in all three domains. The results of the generalized linear model, which assessed the adequacy of each self-care domain based on various domains of social capital, are comprehensively detailed in Table 2. When self-care confidence was incorporated as an additional covariate, the total social capital score retained statistical significance across all domains, indicating that its association with self-care adequacy persisted independently of confidence. However, the magnitude and significance of individual social capital subdomains varied across models.

**Table 2:** Multivariate Analysis of Social Capital and Its Domains on Sufficiency of Heart Failure Self-Care Maintenance Scale, Symptom Perception and Symptom Management: Utilizing generalized linear models with leave-one-out cross-validation for internal validation.

Outcome	Predictors				Primary Models			Secondary Models		
					β Intercept (SE)	Odds Ratio (β Exponential)	*p-*value^1^	β Intercept (SE)	Odds Ratio (β Exponential)	*p*-value
**Maintenance Behaviors**	**Total Social Capital Score**				0.056 (0.014)	1.057 (1.029–1.086)	<0.001	0.050 (0.015)	1.051 (1.021–1.082)	0.001
		Self-care Confidence			–	–	–	1.390 (0.391)	4.016 (1.866–8.642)	0.001
		Other Covariates^2^								
			*Rural vs. Urban Residential*		−1.704 (0.578)	0.182 (0.059–0.565)	0.003	−1.960 (0.605)	0.141 (0.043–0.461)	0.001
			*Waterpipe Addiction*		−1.306 (0.712)	0.271 (0.067–1.094)	0.067	−1.330 (0.719)	0.264 (0.065–1.082)	0.064
			*Smoke Addiction*		−0.420 (0.404)	0.657 (0.298–1.451)	0.299	−0.602 (0.433)	0.548 (0.235–1.278)	0.164
			*Married vs Unmarried*		−0.774 (0.518)	0.461 (0.167–1.275)	0.136	−0.847 (0.544)	0.429 (0.148–1.245)	0.119
	**Community Participation Score**				0.025 (0.008)	1.025 (1.008–1.042)	0.003	0.026 (0.009)	1.026 (1.008–1.045)	0.004
		Self-care Confidence			–	–	–	1.642 (0.391)	5.167 (2.403–11.112)	<0.001
		Other Covariates^2^								
			*Rural vs. Urban Residential*		−1.655 (0.554)	0.191 (0.065–0.566)	0.003	−1.983 (0.591)	0.138 (0.043–0.439)	0.001
			*Waterpipe Addiction*		−1.147 (0.696)	0.318 (0.081–1.242)	0.099	−1.301 (0.738)	0.272 (0.064–1.157)	0.078
			*Smoke Addiction*		−0.375 (0.390)	0.687 (0.320–1.477)	0.337	−0.638 (0.427)	0.528 (0.229–1.220)	0.135
			*Married vs Unmarried*		−0.519 (0.491)	0.595 (0.227–1.559)	0.291	−0.688 (0.527)	0.503 (0.179–1.412)	0.192
	**Pro-Activity in Social Score**				0.026 (0.010)	1.027 (1.008–1.046)	0.005	0.018 (0.010)	1.018 (0.998–1.038)	0.072
		Self-care Confidence			–	–	–	1.395 (0.378)	4.034 (1.922–8.467)	<0.001
		Other Covariates^2^								
			*Rural vs. Urban Residential*		−1.426 (0.550)	0.240 (0.082–0.706)	0.009	−1.682 (0.576)	0.186 (0.060–0.575)	0.004
			*Waterpipe Addiction*		−1.242 (0.690)	0.289 (0.075–1.117)	0.072	−1.281 (0.715)	0.278 (0.068–1.127)	0.073
			*Married vs Unmarried*		−0.662 (0.488)	0.516 (0.198–1.344)	0.175	−0.646 (0.514)	0.524 (0.191–1.434)	0.208
	**Feeling of Trust and Safety Score**				0.020 (0.009)	1.020 (1.002–1.037)	0.027	0.014 (0.009)	1.014 (0.996–1.033)	0.121
		Self-care Confidence			–	–	–	1.456 (0.371)	4.287 (2.073–8.867)	<0.001
		Other Covariates^2^								
			*Rural vs. Urban Residential*		−1.442 (0.534)	0.236 (0.083–0.673)	0.007	−1.675 (0.561)	0.187 (0.062–0.563)	0.003
			*Waterpipe Addiction*		−1.008 (0.687)	0.365 (0.095–1.402)	0.142	−1.093 (0.710)	0.335 (0.083–1.349)	0.124
	**Family and Friends Connections Score**				0.021 (0.007)	1.022 (1.007–1.036)	0.003	0.019 (0.008)	1.020 (1.004–1.035)	0.012
		Self-care Confidence			–	–	–	1.522 (0.383)	4.579 (2.164–9.692)	<0.001
		Other Covariates^2^								
			*Rural vs. Urban Residential*		−1.501 (0.545)	0.223 (0.077– 0.648)	0.006	−1.785 (0.576)	0.168 (0.054–0.520)	0.002
			*Waterpipe Addiction*		−1.209 (0.699)	0.299 (0.076–1.176)	0.084	−1.264 (0.711)	0.283 (0.070–1.139)	0.076
			*Smoke Addiction*		−0.367 (0.394)	0.693 (0.320–1.500)	0.352	−0.567 (0.432)	0.567 (0.243–1.323)	0.189
			*Married vs Unmarried*		−0.452 (0.493)	0.636 (0.242–1.674)	0.359	−0.582 (0.532)	0.559 (0.197–1.585)	0.274
	**Neighborhood Connections Score**				0.032 (0.009)	1.032 (1.015– 1.050)	<0.001	0.027 (0.009)	1.028 (1.010–1.046)	0.003
		Self-care Confidence			–	–	–	1.428 (0.387)	4.169 (1.952–8.908)	<0.001
		Other Covariates^2^								
			*Rural vs. Urban Residential*		−1.752 (0.567)	0.173 (0.057–0.528)	0.002	−2.019 (0.598)	0.133 (0.041–0.429)	0.001
			*Waterpipe Addiction*		−1.337 (0.721)	0.263 (0.064–1.079)	0.064	−1.346 (0.724)	0.260 (0.063–1.075)	0.063
			*Smoke Addiction*		−0.366 (0.399)	0.693 (0.317–1.516)	0.359	−0.524 (0.428)	0.592 (0.256–1.370)	0.221
			*Married vs Unmarried*		−0.475 (0.496)	0.622 (0.235–1.644)	0.338	−0.621 (0.532)	0.537 (0.190–1.524)	0.243
	**Work Connections among Employed Patients**				0.035 (0.011)	1.035 (1.013–1.058)	0.001	0.033 (0.012)	1.033 (1.010–1.058)	0.006
		Self-care Confidence			–	–	–	1.935 (0.604)	6.922 (2.120–22.601)	0.001
		Other Covariates^2^								
			*Waterpipe Addiction*		1.898 (1.351)	6.670 (0.472–94.235)	0.160	1.989 (1.488)	7.310 (0.396–135.034)	0.181
			*Smoke Addiction*		−0.902 (0.646)	0.406 (0.114–1.440)	0.163	−1.126 (0.727)	0.324 (0.078–1.349)	0.121
			*Married vs Unmarried*		−1.134 (0.735)	0.322 (0.076–1.359)	0.123	−1.130 (0.770)	0.323 (0.071–1.463)	0.143
			*Number of Years being under Surveillance of Cardiologist*		0.046 (0.032)	1.047 (0.984–1.115)	0.145	0.046 (0.038)	1.047 (0.972–1.128)	0.222
			*Education Level*		−0.979 (0.529)	0.376 (0.133–1.059)	0.064	−1.057 (0.582)	0.347 (0.111–1.088)	0.070
			*HFrEF vs. HFmrEF/HFpEF*		−1.249 (0.860)	0.287 (0.053–1.546)	0.146	−1.748 (0.982)	0.174 (0.025–1.194)	0.075
**Symptom Perception**	**Total Social Capital Score**				0.046 (0.016)	1.047 (1.015–1.081)	0.004	0.041 (0.019)	1.042 (1.004–1.081)	0.029
		Self-care Confidence			–	–	–	2.287 (0.576)	9.841 (3.181–30.445)	<0.001
		Other Covariates^2^								
			*Married vs Unmarried*		0.870 (0.743)	2.387 (0.556–10.250)	0.242	1.234 (0.832)	3.437 (0.673–17.539)	0.138
			*Smoking*		−0.551 (0.528)	0.576 (0.205–1.620)	0.296	−0.742 (0.581)	0.476 (0.153–1.486)	0.200
			*Number of Years being under Surveillance of Cardiologist*		0.061 (0.028)	1.062 (1.006–1.122)	0.028	0.069 (0.032)	1.072 (1.007–1.141)	0.029
			*Number of Comorbidities*							
				*1vs 0*	1.826 (0.704)	6.206 (1.561– 24.667)	0.010	1.921 (0.758)	6.828 (1.545–30.168)	0.011
				*2vs 0*	1.838 (0.746)	6.282 (1.456–27.100)	0.014	2.123 (0.812)	8.357 (1.701–41.062)	0.009
				*3vs 0*	1.002 (0.819)	2.723 (0.547–13.554)	0.221	1.725 (0.959)	5.615 (0.857–36.782)	0.072
				*4vs 0*	1.489 (0.859)	4.434 (0.823–23.887)	0.083	1.221 (0.916)	3.392 (0.563–20.435)	0.183
				*5vs 0*	3.107 (1.572)	22.355 (1.026– 487.001)	0.048	4.610 (1.677)	100.496 (3.758– 2687.415)	0.006
	**Community Participation Score**				0.021 (0.010)	1.021 (1.002 – 1.041)	0.027	0.025 (0.011)	1.025 (1.003–1.048)	0.028
		Self-care Confidence			–	–	–	2.473 (0.579)	11.862 (3.812–36.916)	<0.001
		Other Covariates^2^								
			*Smoke Addiction*		−0.557 (0.527)	0.573 (0.204–1.611)	0.291	−0.829 (0.586)	0.436 (0.138–1.377)	0.157
			*Married vs Unmarried*		1.005 (0.720)	2.731 (0.666–11.196)	0.163	1.368 (0.834)	3.926 (0.766–20.119)	0.101
			*Number of Years being under Surveillance of Cardiologist*		0.052 (0.027)	1.053 (0.999–1.111)	0.053	0.058 (0.033)	1.060 (0.993–1.131)	0.080
			*Number of Comorbidities*							
				*1vs 0*	1.862 (0.722)	6.434 (1.563–26.478)	0.010	2.161 (0.815)	8.680 (1.756–42.912)	0.008
				*2vs 0*	1.887 (0.757)	6.601 (1.497–29.103)	0.013	2.306 (0.854)	10.030 (1.882–53.468)	0.007
				*3vs 0*	1.004 (0.824)	2.729 (0.543–13.711)	0.223	1.928 (0.990)	6.878 (0.988–47.905)	0.051
				*4vs 0*	1.538 (0.857)	4.657 (0.868–24.981)	0.073	1.283 (0.944)	3.609 (0.567–22.959)	0.174
				*5vs 0*	2.644 (1.551)	14.066 (0.672–294.263)	0.088	4.447 (1.670)	85.396 (3.238–2251.959)	0.008
	**Pro-Activity in Social Score**				0.041 (0.013)	1.041 (1.016–1.068)	0.001	0.031 (0.013)	1.031 (1.005–1.058)	0.021
		Self-care Confidence			–	–	–	2.161 (0.574)	8.677 (2.815–26.744)	<0.001
		Other Covariates^2^								
			*Smoke Addiction*		−0.582 (0.522)	0.559 (0.201–1.553)	0.265	−0.827 (0.578)	0.437 (0.141–1.358)	0.152
			*Number of Years being under Surveillance of Cardiologist*		0.057 (0.028)	1.059 (1.002–1.119)	0.043	0.062 (0.032)	1.064 (1.001–1.132)	0.048
			*Number of Comorbidities*							
				*1vs 0*	1.625 (0.689)	5.077 (1.316–19.585)	0.018	1.617 (0.720)	5.040 (1.228–20.683)	0.025
				*2vs 0*	1.570 (0.719)	4.806 (1.175–19.652)	0.029	1.735 (0.761)	5.668 (1.275–25.191)	0.023
				*3vs 0*	0.725 (0.809)	2.065 (0.423–10.081)	0.370	1.432 (0.926)	4.188 (0.682–25.721)	0.122
				*4vs 0*	1.688 (0.878)	5.410 (0.968–30.232)	0.054	1.406 (0.918)	4.079 (0.674–24.683)	0.126
				*5vs 0*	2.964 (1.553)	19.370 (0.923–0.923)	0.056	4.343 (1.633)	76.973 (3.133–1890.948)	0.008
	**Feeling of Trust and Safety Score**				0.029 (0.012)	1.029 (1.006–1.053)	0.015	0.021 (0.012)	1.022 (0.997–1.047)	0.082
		Self-care Confidence			–	–	–	2.382 (0.582)	10.827 (3.458–33.904)	<0.001
		Other Covariates^2^								
			*Opioid Addiction*		−0.738 (0.752)	0.478 (0.109–2.088)	0.326	−1.197 (0.833)	0.302 (0.059–1.546)	0.151
			*Care Setting*		−0.919 (0.736)	0.399 (0.094–1.688)	0.212	−1.306 (0.878)	0.271 (0.048–1.515)	0.137
			*Married vs Unmarried*		0.881 (0.726)	2.414 (0.581–10.026)	0.225	1.196 (0.827)	3.306 (0.653–16.724)	0.148
			*Number of Years being under Surveillance of Cardiologist*		0.068 (0.028)	1.070 (1.012–1.131)	0.016	0.074 (0.031)	1.077 (1.013–1.146)	0.018
			*Number of Comorbidities*							
				*1vs 0*	1.428 (0.673)	4.170 (1.114–15.600)	0.034	1.427 (0.708)	4.167 (1.040–16.692)	0.044
				*2vs 0*	1.666 (0.736)	5.290 (1.250–22.386)	0.024	1.830 (0.790)	6.233 (1.324–29.335)	0.021
				*3vs 0*	0.677 (0.826)	1.968 (0.390–9.931)	0.413	1.161 (0.953)	3.194 (0.494–20.667)	0.223
				*4vs 0*	1.395 (0.857)	4.035 (0.753–21.631)	0.103	1.125 (0.895)	3.080 (0.533–17.794)	0.209
				*5vs 0*	2.583 (1.565)	13.236 (0.616–284.324)	0.099	4.299 (1.668)	73.631 (2.802–1934.668)	0.010
	**Work Connections among Employed Patients**				0.033 (0.015)	1.033 (1.004–1.063)	0.027	0.036 (0.017)	1.037 (1.004–1.071)	0.029
		Self-care Confidence			–	–	–	3.046 (1.128)	21.039 (2.305–192.054)	0.007
		Other Covariates								
			Smoke Addiction		−1.998 (1.114)	0.136 (0.015–1.204)	0.073	−2.514 (1.223)	0.081 (0.007–0.890)	0.040
**Symptom Management**	**Total Social Capital Score**				0.047 (0.016)	1.048 (1.015–1.081)	0.004	0.040 (0.018)	1.041 (1.004–1.079)	0.028
		Self-care Confidence			–	–	–	2.001 (0.515)	7.400 (2.696–20.313)	<0.001
		Other Covariates^2^								
			*Rural vs Urban Residential*		−0.582 (0.582)	0.559 (0.179–1.748)	0.317	−0.685 (0.618)	0.504 (0.150–1.694)	0.268
			*Education Level*		0.686 (0.546)	1.986 (0.680–5.795)	0.209	0.829 (0.604)	2.291 (0.701–7.485)	0.170
			*Smoke Addiction*		−0.741 (0.519)	0.477 (0.173–1.317)	0.153	−1.022 (0.578)	0.360 (0.116–1.117)	0.077
			*Waterpipe Addiction*		−1.387 (0.879)	0.250 (0.045–1.399)	0.115	−1.378 (0.910)	0.252 (0.042–1.499)	0.130
			*Care Setting*		−1.578 (0.705)	0.206 (0.052–0.821)	0.025	−1.930 (0.799)	0.145 (0.030–0.695)	0.016
			*Number of Years being under Surveillance of Cardiologist*		0.046 (0.027)	1.047 (0.992–1.105)	0.094	0.054 (0.031)	1.056 (0.993–1.122)	0.082
			*Number of Comorbidities*							
				*1vs 0*	0.903 (0.603)	2.467 (0.756–8.050)	0.135	0.777 (0.658)	2.175 (0.599–7.896)	0.238
				*2vs 0*	1.627 (0.639)	5.090 (1.454–17.815)	0.011	1.753 (0.706)	5.770 (1.447–23.006)	0.013
				*3vs 0*	−0.683 (0.853)	0.505 (0.095–2.691)	0.424	−0.457 (1.000)	0.633 (0.089–4.493)	0.647
				*4vs 0*	−0.524 (0.954)	0.592 (0.091–3.838)	0.583	−1.011 (1.016)	0.364 (0.050–2.666)	0.320
				*5vs 0*	1.961 (1.538)	7.107 (0.349–144.845)	0.202	3.036 (1.617)	20.827 (0.875–495.988)	0.060
	**Community Participation Score**				0.020 (0.009)	1.021 (1.002–1.040)	0.029	0.025 (0.011)	1.025 (1.003–1.048)	0.024
		Self-care Confidence			–	–	–	2.219 (0.524)	9.197 (3.294–25.667)	<0.001
		Other Covariates^2^								
			*Rural vs Urban Residential*		−0.659 (0.572)	0.517 (0.169–1.588)	0.250	−0.809 (0.608)	0.445 (0.135–1.465)	0.183
			*Education Level*		0.401 (0.509)	1.493 (0.551–4.047)	0.431	0.724 (0.585)	2.062 (0.655–6.488)	0.216
			*Smoke Addiction*		−0.765 (0.491)	0.465 (0.178–1.218)	0.119	−1.189 (0.559)	0.305 (0.102–0.911)	0.033
			*Waterpipe Addiction*		−1.444 (0.854)	0.236 (0.044–1.260)	0.091	−1.587 (0.930)	0.205 (0.033–1.267)	0.088
			*Care Setting*		−1.377 (0.691)	0.252 (0.065–0.978)	0.046	−1.810 (0.788)	0.164 (0.035–0.766)	0.022
			*Number of Comorbidities*							
				*1vs 0*	0.923 (0.591)	2.518 (0.791–8.011)	0.118	0.985 (0.661)	2.677 (0.733–9.772)	0.136
				*2vs 0*	1.601 (0.623)	4.958 (1.461–16.817)	0.010	1.845 (0.705)	6.326 (1.588–25.193)	0.009
				*3vs 0*	−0.605 (0.817)	0.546 (0.110–2.707)	0.459	−0.112 (0.932)	0.894 (0.144–5.558)	0.904
				*4vs 0*	−0.279 (0.917)	0.757 (0.125–4.567)	0.761	−0.799 (0.973)	0.450 (0.067–3.029)	0.412
				*5vs 0*	1.482 (1.517)	4.400 (0.225–86.004)	0.329	2.837 (1.607)	17.058 (0.731–397.974)	0.078
	**Pro-Activity in Social Score**				0.044 (0.013)	1.045 (1.019–1.073)	0.001	0.037 (0.014)	1.038 (1.009–1.067)	0.009
		Self-care Confidence			–	–	–	1.935 (0.524)	6.927 (2.480–19.348)	<0.001
		Other Covariates^2^								
			*Education Level*		0.936 (0.572)	2.549 (0.831–7.820)	0.102	0.973 (0.618)	2.645 (0.787–8.885)	0.116
			*Smoke Addiction*		−0.771 (0.526)	0.462 (0.165–1.297)	0.143	−1.112 (0.590)	0.329 (0.103–1.045)	0.059
			*Waterpipe Addiction*		−1.439 (0.871)	0.237 (0.043–1.308)	0.099	−1.495 (0.914)	0.224 (0.037–1.346)	0.102
			*Care Setting*		−1.272 (0.663)	0.280 (0.076–1.029)	0.055	−1.649 (0.755)	0.192 (0.044–0.845)	0.029
			*Number of Years being under Surveillance of Cardiologist*		0.049 (0.028)	1.051 (0.994–1.110)	0.079	0.053 (0.031)	1.055 (0.993–1.120)	0.084
			*Number of Comorbidities*							
				*1vs 0*	0.781 (0.599)	2.184 (0.675–7.070)	0.192	0.775 (0.644)	2.171 (0.615–7.663)	0.229
				*2vs 0*	1.578 (0.641)	4.846 (1.379–17.025)	0.014	1.828 (0.713)	6.222(1.537–25.190)	0.010
				*3vs 0*	−0.813 (0.824)	0.444 (0.088–2.230)	0.324	−0.469 (0.955)	0.626 (0.096–4.068)	0.624
				*4vs 0*	−0.456 (0.977)	0.634 (0.093–4.305)	0.641	−0.821 (1.027)	0.440 (0.059–3.291)	0.424
				*5vs 0*	0.049 (0.028)	1.051 (0.994–1.110)	0.079	3.059 (1.593)	21.305 (0.938–483.739)	0.055
	**Feeling of Trust and Safety Score**				0.018 (0.011)	1.018 (0.997–1.040)	0.097	0.010 (0.012)	1.010 (0.987–1.033)	0.389
		Self-care Confidence			–	–	–	2.128 (0.520)	8.395 (8.395–23.261)	<0.001
		Other Covariates^2^								
			*Age*		0.019 (0.020)	1.019 (0.979–1.061)	0.349	0.030 (0.023)	1.031 (0.985–1.078)	0.192
			*Smoke Addiction*		−0.456 (0.491)	0.634 (0.242–1.659)	0.353	−0.729 (0.546)	0.482 (0.165–1.407)	0.182
			*Waterpipe Addiction*		−1.148 (0.849)	0.317 (0.060–1.677)	0.177	−1.231 (0.899)	0.292 (0.050–1.700)	0.171
			*Care Setting*		−1.509 (0.686)	0.221 (0.058–0.848)	0.028	−1.864 (0.774)	0.155 (0.034–0.707)	0.016
			*Number of Years being under Surveillance of Cardiologist*		0.041 (0.027)	1.042 (0.988–1.098)	0.132	0.043 (0.030)	1.044 (0.985–1.107)	0.150
			*Number of Comorbidities*							
				*1vs 0*	0.658 (0.570)	1.931 (0.632–5.898)	0.248	0.564 (0.602)	1.757 (0.540–5.717)	0.349
				*2vs 0*	1.515 (0.605)	4.547 (1.389–14.883)	0.012	1.677 (0.676)	5.348 (1.421–20.134)	0.013
				*3vs 0*	−0.626 (0.803)	0.535 (0.111–2.581)	0.436	−0.315 (0.920)	0.730 (0.120–4.431)	0.732
				*4vs 0*	−0.422 (0.921)	0.656 (0.108–3.986)	0.647	−1.039 (0.989)	0.354 (0.051–2.457)	0.293
				*5vs 0*	1.617 (1.490)	5.035 (0.271–93.481)	0.278	2.944 (1.567)	18.984 (0.880– 409.742)	0.060
	**Family and Friends Connections Score**				0.015 (0.009)	1.015 (0.998–1.033)	0.082	0.015 (0.010)	1.015 (0.996–1.035)	0.119
		Self-care Confidence			–	–	–	2.203 (0.525)	9.051 (3.233–25.343)	<0.001
		Other Covariates^2^								
			*Age*		0.025 (0.021)	1.025 (0.984–1.068)	0.233	0.035 (0.024)	1.035 (0.989–1.084)	0.142
			*Smoke Addiction*		−0.471 (0.490)	0.624 (0.239–1.632)	0.337	−0.822 (0.559)	0.439 (0.147–1.315)	0.141
			*Waterpipe Addiction*		−1.294 (0.850)	0.274 (0.052–1.451)	0.128	−1.315 (0.887)	0.269 (0.047–1.526)	0.138
			*Care Setting*		−1.463 (0.680)	0.232 (0.061–0.877)	0.031	−1.936 (0.777)	0.144 (0.031–0.662)	0.013
			*Number of Years being under Surveillance of Cardiologist*		0.033 (0.027)	1.033 (0.980–1.090)	0.226	0.040 (0.030)	1.040 (0.981–1.103)	0.186
			*Number of Comorbidities*							
				*1vs 0*	0.692 (0.569)	1.998 (0.655–6.092)	0.224	0.700 (0.607)	2.014 (0.613–6.621)	0.249
				*2vs 0*	1.613 (0.606)	5.017 (1.529–16.467)	0.008	1.863 (0.694)	6.444 (1.655–25.089)	0.007
				*3vs 0*	−0.572 (0.813)	0.564 (0.115–2.779)	0.482	−0.148 (0.940)	0.863 (0.137–5.443)	0.875
				*4vs 0*	−0.519 (0.925)	0.595 (0.097–3.644)	0.575	−1.122 (0.999)	0.326 (0.046–2.306)	0.261
				*5vs 0*	2.367 (1.566)	10.666 (0.495–229.767)	0.131	3.821 (1.692)	45.672 (1.658–1257.823)	0.024
	**Neighborhood Connections Score**				0.028 (0.010)	1.028 (1.008–1.049)	0.006	0.024 (0.012)	1.024 (1.001–1.048)	0.044
		Self-care Confidence			–	–	–	2.216 (0.544)	9.167 (3.159–26.602)	<0.001
		Other Covariates^2^								
			*Age*		0.023 (0.021)	1.023 (0.981–1.066)	0.284	0.027 (0.024)	1.027 (0.981–1.076)	0.256
			*Rural vs Urban Residential*		−0.677 (0.593)	0.508 (0.159–1.625)	0.254	−0.823 (0.639)	0.439 (0.126–1.537)	0.198
			*Smoke Addiction*		−0.706 (0.520)	0.493 (0.178–1.367)	0.174	−0.997 (0.580)	0.369 (0.118–1.149)	0.085
			*Waterpipe Addiction*		−1.373 (0.894)	0.253 (0.044–1.462)	0.125	−1.300 (0.890)	0.273 (0.048–1.560)	0.144
			*Care Setting*		−1.983 (0.777)	0.138 (0.030–0.631)	0.011	−2.588 (0.889)	0.075 (0.013–0.429)	0.004
			*Income Level*							
				*Middle vs. Low*	−0.505 (0.529)	0.603 (0.214–1.703)	0.340	−1.052 (0.623)	0.349 (0.103–1.184)	0.091
				*High vs. Low*	−0.556 (1.296)	0.574 (0.045–7.269)	0.668	−0.690 (1.343)	0.501 (0.036–6.979)	0.607
			*Number of Years being under Surveillance of Cardiologist*		0.039 (0.028)	1.040 (0.984–1.099)	0.167	0.051 (0.032)	1.052 (0.989–1.120)	0.108
			*Number of Comorbidities*							
				*1vs 0*	0.856 (0.596)	2.354 (0.732–7.570)	0.151	0.612 (0.633)	1.844 (0.533–6.382)	0.334
				*2vs 0*	1.550 (0.634)	4.714 (1.361–16.331)	0.014	1.592 (0.699)	4.914 (1.248–19.346)	0.023
				*3vs 0*	−0.700 (0.837)	0.497 (0.096–2.562)	0.403	−0.500 (0.959)	0.607 (0.093–3.975)	0.602
				*4vs 0*	−0.469 (0.952)	0.626 (0.097–4.046)	0.622	−1.083 (1.046)	0.339 (0.044–2.630)	0.300
				*5vs 0*	2.309 (1.626)	10.063 (0.415–243.815)	0.156	3.312 (1.711)	27.453 (0.959–785.843)	0.053

1 All p-values less than 0.05 are considered statistically significant.

2 Additional covariates incorporated within the models include: age, sex, residential area, care setting (hospital or clinic), perceived income level, education level, addiction to tobacco, waterpipe, opioids, number of comorbidities, heart failure subtype, number of years under cardiologist’s surveillance, and left ventricular ejection fraction. Abbreviations. SE: standard error, HFrEF: heart failure with reduced ejection fraction; HFpEF: heart failure with preserved ejection fraction, HFmrEF: heart failure with mildly reduced ejection fraction.

### 3.2 Role of community participation in self-care of heart failure domains

**Fig. 2:**
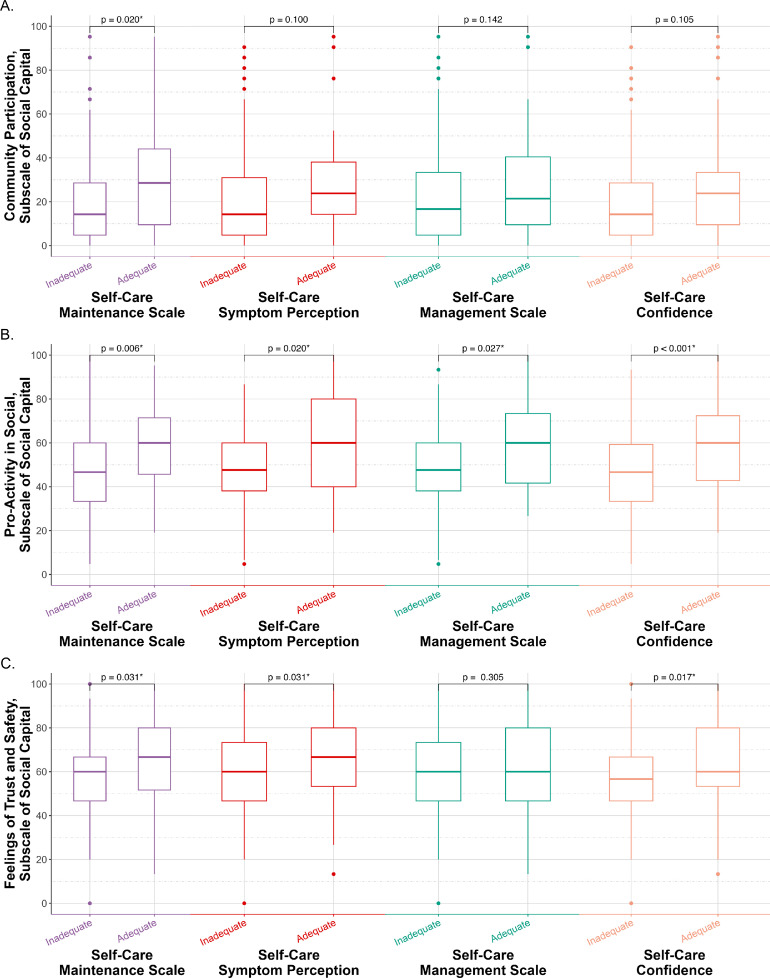
**Associations between social capital subscales and self-care domains in patients with heart failure. (A)** Differences in *Community Participation* subscale scores of social capital (standardized) between patients with adequate *versus* inadequate self-care scores across three domains of the Self-Care of Heart Failure index: self-care maintenance, symptom perception, and symptom management, as well as confidence. **(B)** Differences in *Pro-Activity in Social* subscale scores of social capital between patients with adequate *versus* inadequate scores in each self-care domain. **(C)** Differences in *Social Safety and Trust Feeling* subscale scores of social capital between patients with adequate *versus* inadequate scores in each self-care domain. Box plots display medians, interquartile ranges, and outliers; *p-*values are from group comparisons.

Regarding the first domain of social capital, community participation scored higher among heart failure patients with adequate maintenance behaviors than those with inadequate maintenance in both univariate and multivariate analyses, as shown in Fig. 2A and Table 2. This association remained significant after adjusting for self-care confidence, reflecting that the association between community participation and maintenance behaviors was not explained by confidence levels. Furthermore, heart failure patients with adequate symptom perception and management likewise demonstrated higher community participation scores, as illustrated in Fig. 2A. Despite the lack of statistical significance in univariate analysis, multivariate models identified community participation as a significant independent predictor of both symptom perception and symptom management, again unaffected by the inclusion of self-care confidence (Table 2). Finally, although patients with higher self-care confidence tended to report higher community participation, the observed difference did not reach statistical significance in either univariate (*p* = 0.105) or multivariate analyses (*p* = 0.339).

### 3.3 Role of social proactivity in self-care of heart failure domains

Fig. 2B illustrates the role of proactivity in social contexts among heart failure patients with varying levels of self-care behavior adequacy and confidence. Heart failure Patients with adequate maintenance behaviors, symptom perception, and symptom management demonstrated significantly higher proactivity scores compared to those with inadequate self-care domains (all *p* < 0.05). This was further validated through multivariate analysis, accounting for other sociodemographic contributors, as detailed in the primary models in Table 2. In secondary models by incorporating self-care confidence, the association between proactivity and maintenance behaviors was attenuated, indicating that confidence partly accounted for this relationship, whereas the associations with symptom perception and symptom management remained independent of confidence. Heart failure patients with sufficient self-care confidence had higher proactivity scores than those with low self-care confidence. After controlling for confounders, the adjusted OR of proactivity for adequacy of confidence was recorded at 1.038 (CI 95 %: 1.017–1.060, *p* < 0.001). These findings emphasize the important role of proactivity in promoting effective self-care behaviors and confidence among heart failure patients.

### 3.4 Role of feeling of trust and safety in self-care of heart failure domains

Fig. 2C implies the significance of trust and safety feelings within society across various dimensions of self-care behaviors and self-care confidence. Notably, the level of perceived trust and safety was significantly higher among heart failure patients exhibiting adequate maintenance behaviors and symptom perception, consistent with the primary models presented in Table 2. In multivariate secondary models, the associations with both maintenance behaviors and symptom perception were attenuated after incorporating self-care confidence, suggesting that confidence accounts for part of these relationships. Additionally, heart failure patients possessing adequate self-care confidence exhibited a higher sense of safety and trust, compared to those with inadequate self-care confidence, with *p-*values of 0.017 in univariate analysis and 0.014 in multivariate analysis (OR: 1.022, 95 % CI: 1.005–1.041). Overall, these findings indicated that perceived feelings of trust and safety within society may support self-care maintenance and symptom perception, in part through their association with self-care confidence.

### 3.5 Role of neighborhood connections in self-care of heart failure domains

**Fig. 3:**
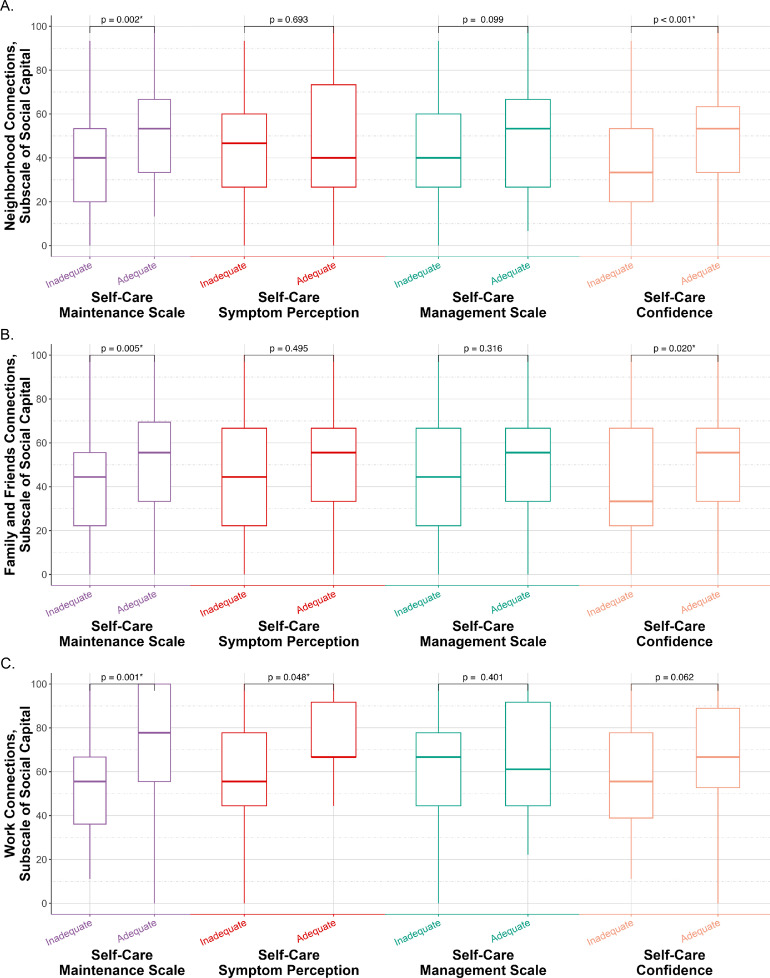
**Associations between additional social capital subscales and self-care domains in patients with heart failure. (A)** Differences in *Neighborhood Connection* subscale scores of social capital between patients with adequate *versus* inadequate self-care scores across three domains of the Self-Care of Heart Failure index: self-care maintenance, symptom perception, and symptom management, as well as confidence. **(B)** Differences in *Family and Friends Connection* subscale scores of social capital between patients with adequate *versus* inadequate scores in each self-care domain. **(C)** Differences in *Work Connection* subscale scores of social capital between employed patients with adequate *versus* inadequate scores in each self-care domain. Box plots display medians, interquartile ranges, and outliers; *p*-values are from group comparisons.

As seen in Fig. 3A, heart failure patients with adequate maintenance behaviors scored higher in neighborhood connections compared to patients with inadequate maintenance. Multivariate analysis also showed the significance of higher neighborhood connections in maintenance behaviors and symptom management, which remained significant after adding self-care confidence (all *p-*values<0.05). This indicates that the positive effects of neighborhood connections on these two self-care domains were independent of confidence. Higher neighborhood connection was linked to adequate self-care confidence, as heart failure patients with sufficient self-care confidence exhibited higher neighborhood connection scores, with an adjusted OR of 1.028 (95 % CI: 1.011–1.046, *p* = 0.001).

### 3.6 Role of family and friends connections in self-care of heart failure domains

Family and Friends Connections were associated with adequacy of maintenance behaviors and self-care confidence in both univariate and multivariate analyses of Fig. 3B and Table 2. Heart failure patients with adequate maintenance behaviors had stronger family and friend relationships than those with inadequate maintenance behaviors, which remained significant after accounting for self-care confidence, indicating independence of this relation from confidence. Furthermore, these connections were similarly higher among heart failure patients with adequate self-care confidence compared to patients with inadequate confidence. However, this difference did not maintain statistical significance after controlling for confounding variables, with the adjusted *p-*value being 0.068. These findings suggest that family and friend connections may play a supportive role in reinforcing maintenance behaviors through self-care confidence.

### 3.7 Role of work connections in self-care of heart failure domains

Work Connections emerged as a significant factor influencing both maintenance behaviors and symptom perception. Notably, heart failure patients having adequate maintenance behaviors or adequate symptom perception reported higher work connections in both univariate and multivariate analyses, demonstrated in Fig. 3C and Table 2. Furthermore, these associations remained significant in secondary models, revealing that the effects of work connections on maintenance behaviors and symptom perception were independent of self-care confidence. Heart failure patients with adequate self-care confidence also exhibited higher work connections, which reached statistical significance after controlling for confounding variables (OR: 1.023, 95 % CI: 1.003–1.043, *p* = 0.022).

### 3.8 Role of tolerance of diversity in self-care of heart failure domains

The distribution of tolerance of diversity is presented in **Supplementary Material**, *Figure S3B*. Scores for this domain were consistent across heart failure patients, regardless of the adequacy of their maintenance behaviors, symptom management, or self-care confidence. All median values were recorded at 50 (IQR: 33.33–66.67), with all *p-*value*s* exceeding 0.05, indicating non-significant differences. Regarding symptom perception, tolerance of diversity was higher among patients with adequate symptom perception, with a median of 66.67 (IQR: 33.33–66.67) *versus* 50 (IQR: 33.33–66.67). This difference approached but did not reach statistical significance in multivariate analysis (*p*-value=0.064).

### 3.9 Role of value of life in self-care of heart failure domains

The distribution of the last remaining domain of social capital is presented in **Supplementary Material**, *Figure S3C*. Regarding the Value of Life, although heart failure patients with adequate maintenance behaviors, symptom perception, or symptom management scored higher, with all medians being 66.67 (IQR: 33.33–83.33) *versus* 50 (IQR: 33.33–66.67), these differences did not reach statistical significance in univariate analysis (all *p-*values>0.05). In adjusted models, *p*-values approached but did not cross the significance threshold for maintenance (*p* = 0.067), symptom perception (*p* = 0.064), and symptom management (*p* = 0.076). These findings suggest that, while Value of Life may trend higher among patients with more adequate self-care, its associations with specific self-care domains were not statistically supported.

## 4 Discussion

In this multicenter sample of heart failure patients, we observed a high prevalence of inadequate self-care maintenance (59.24 %), symptom perception (76.43 %), symptom management (71.43 %), and self-care confidence (52.23 %). In comparison, Schäfer-Keller et al.’s investigation on hospitalized heart failure patients reported 76 % inadequate self-care maintenance, 90 % inadequate symptom management, and 39 % inadequate self-care confidence. We showed better maintenance and symptom management but worse confidence compared to the results of Schäfer-Keller et al. (2021). This difference could be attributed to the fact that most of our participants were heart failure patients from outpatient clinics rather than inpatients. However, both studies show that inadequate self-care is more prevalent, underscoring the need to identify the main barriers to adequate self-care, as it can reduce hospitalization burdens, mortality rates, and enhance patients’ quality of life (Heidenreich et al., 2022; McDonagh et al., 2021).

We revealed the association between higher social capital and better self-care of heart failure, including maintenance behaviors, symptom perception, and management. To the best of our knowledge, this is the first study investigating this association. A recent systematic review conducted by Kleman et al. (2024), encompassing 52 articles, has elucidated various individual and system-related factors impacting heart failure self-care. According to Bronfenbrenner’s Socioecological Model (Bronfenbrenner, 1979), system-related factors can be classified into three levels: (I) The microsystem-level factors, which includes influences from family, peers, friends, extended family, and neighborhood; (II) The exosystem-level factors, which pertains to the work environment, mass media, healthcare organizations, social organizations, and religious organizations; and (III) The macrosystem-level factors, which encompasses broader societal elements, such as laws, culture, history, social conditions, and the economic system. Kleman et al. (2024) pointed out in their systematic review that existing literature has thoroughly examined 35 individual-level factors, while only 11 microsystem-level factors have been studied. Notably, there has been a lack of studies exploring exosystem or macrosystem factors contributing to heart failure self-care. Prior researchers have focused mainly on social support, which is a microsystem-level system-related factor (Kleman, 2024).

In current literature, two systematic reviewers have compiled previous investigations on the role of social support in self-care of heart failure (Babygeetha and Devineni, 2024; Graven and Grant, 2014). Early studies, such as those by Heo et al. (2008) and Sayers et al. (2008), proposed non-significant relationships between social support and various domains of self-care behaviors. Conversely, later studies have provided clearer insights into both direct and indirect effects on these domains, with the latter being mediated by self-care confidence (Dickson et al., 2013; Gallagher et al., 2011; Salyer et al., 2012; Yunus and Sharoni, 2016). The disparities in results may be attributed to factors such as small sample sizes, patient selection from outpatient departments *versus* hospitals, and the use of older versions of self-care questionnaires that encompass different questions and aspects.

Social support encompasses emotional, instrumental, informational, and affirmational support that patients perceive from their immediate environment, which includes relatives, friends, and neighbors (members of their closest social network) (White-Williams et al., 2020). In contrast, social capital provides a more comprehensive assessment of socioecological factors by measuring community participation, proactivity in social environments, feelings of trust and safety in society, neighborhood connections, relationships with family and friends, tolerance of diversity, the value of life, and work connections (Kawachi and Berkman, 2014). Measurement of social capital encompasses all three levels of microsystem, exosystem, and macrosystem factors, particularly those outside the immediate family structure (Bryant, 2012; Dolan, 2022; Ferlander, 2007). Thus, we have provided potentially valuable insights into new aspects of social determinants, notably community participation and proactive engagement, that influence self-care among individuals with heart failure.

Zhang et al. (2023) and Mei et al. (2019) unveiled the significant direct and indirect effects of social support on both maintenance and symptom management domains of self-care. In our study, social capital had positive relations to all three domains of self-care, including self-care maintenance, symptom perception, and management, all of which were independent of self-care confidence. Prior researchers highlighted that social support perceived from family, friends, and significant others influenced self-care confidence, while emotional and instrumental support enhanced the maintenance domain (Sayers et al., 2008). In our study, the strengths of family and friends’ connections enhanced maintenance behaviors, while neighborhood connections increased both maintenance and symptom management domains. Notably, these relations for both familial/friend and neighborhood connections remained significant, independent of confidence in our study. Empirical researchers studying other chronic diseases have also mentioned neighborhood social capital as a mechanism for self-management. For example, in a longitudinal study, Waverijn et al. (2014) demonstrated that both individual and neighborhood social capital were associated with improved self-rated health. Additionally, Waverijn et al. (2017) conducted mediation analyses on a large cohort of patients with chronic illnesses and found that the relationship between neighborhood social capital and self-rated health was mediated by improvements in self-management.

We showed that higher community participation was associated with better self-care maintenance, symptom perception, and symptom management, independent of confidence. In the Onyx & Bullen (2000) framework, this domain reflects how much individuals invest time and effort in collective local activities and civic involvement, such as volunteering, membership in community groups, participating in neighborhood projects, or contributing to local action. Although prior researchers have not directly examined this social capital domain among patients with heart failure, evidence from other chronic conditions supports its relevance. For example, Christian et al. (2020) found that active civic involvement, a key component of social capital, was positively associated with subjective well-being among older adults with non-communicable diseases across six low- and middle-income countries.

Another relatively under-explored domain of social capital examined in this study was proactivity in a social context, which reflects the belief in one’s power to influence the social environment. In Onyx & Bullen’s (2000) model, proactivity captures an individual’s capacity to initiate social action, contribute to community decision-making, and engage in collective problem-solving. This dimension of social capital may enhance self-care by enabling patients to actively mobilize and shape social networks that support health. Empirical evidence from chronic illness contexts supports this claim. For instance, Vassilev et al.’s (2011) realist review highlighted that personal social networks and the processes of “network negotiation” and “collective efficacy”, processes that mirror proactivity, are essential for chronic illness self-management. In our study, higher proactivity was associated with adequate maintenance behaviors, although this relationship was partly explained by self-care confidence. In contrast, the associations of proactivity with symptom perception and symptom management remained significant after incorporating confidence, indicating that, for these domains, proactivity exerted an effect independent of confidence.

In addition to the previously reported effect of employment status on self-care behaviors (Rørth et al., 2016), we highlighted that stronger connections with coworkers, an exosystem-level factor, were associated with better maintenance behaviors and symptom perception. These associations remained significant after incorporating self-care confidence, indicating that work-related social ties can influence these self-care domains independently of confidence. Prior researchers showed that workplace support can improve chronic disease management through informational sharing, role flexibility, and collective norms (Rørth et al., 2016). Feeling of trust and safety in the community also influenced maintenance behaviors and symptom perception. However, these associations were no longer significant once self-care confidence was included in the models, suggesting that their effects were mediated through confidence.

### 4.1 Implications for clinical practice

Our findings can be implemented into heart failure self-care strategies by both clinicians and nurses. Health professionals can routinely assess social capital profiles of heart failure patients, especially regarding community participation, proactivity, neighborhood connections, and co-worker relations, to identify patients who may lack social resources that support self-care (Ehsan et al., 2019; Villalonga-Olives et al., 2018). Interventions should not solely focus on individual motivation or self-efficacy but also aim to strengthen social environments around heart failure patients (Lee et al., 2020). For instance, nurse-led programs could facilitate peer support groups, neighborhood self-management circles, or “social prescribing” to civic organizations (Office of the Surgeon General, 2023). For employed patients, developing self-care advocates or peer networks within workplaces may reinforce daily maintenance behaviors and symptom awareness (Rørth et al., 2016). Because confidence mediated some but not all associations between social capital domains and self-care, interventions should simultaneously build social connections and boost patient confidence through empowerment workshops or collective efficacy activities (Coll-Planas et al., 2017; Villalonga-Olives et al., 2018). Ultimately, harnessing social capital may reduce the clinical burden, prevent rehospitalizations, and improve quality of life in patients with heart failure.

### 4.2 Strengths and limitations

Our study is distinct from previous research in several ways: (I) it provides insights into novel aspects of social relations not represented in existing social support questionnaires; (II) although previous researchers have reported relatively high social support among heart failure patients (Årestedt et al., 2013; Gallagher et al., 2011), we found low to median scores for social capital, thereby highlighting a critical yet underrecognized gap in the broader social determinants influencing heart failure self-care, which should be considered when designing interventions; (III) to our knowledge, this study is unique in employing multicenter, stratified multistage sampling, whereas most other studies have implemented convenience sampling and single-center patient recruitment (Kleman, 2024); (IV) Including both inpatients and outpatients heart failure patients across public and private clinics, which are stratified by care setting and healthcare system type, extends representativeness and external validity of our study. (V) Moreover, we addressed methodological limitations of prior works, which were largely restricted to single-center designs and focused exclusively on either hospitalized or outpatient populations (Kleman, 2024). By incorporating a broader and more representative patient sample and assessing multiple domains of social capital, we have offered a more comprehensive and nuanced understanding of how social capital influences self-care behaviors in heart failure.

We recognize that the cross-sectional study design has inherent limitations, as it is unable to evaluate temporal or causal inferences. Longitudinal or interventional studies are needed to confirm directionality and causality. Although this study was conducted across multiple centers and included both hospitalized and outpatient populations, the overall sample size was modest. While this may limit the generalizability of our findings, the sample size was determined based on statistical calculations and was sufficient to detect the expected correlations with adequate statistical power. The sample was exclusively drawn from Persian-speaking patients from southern Iran, thereby constraining the generalizability of the findings to other cultural, linguistic, or healthcare settings. Information about both self-care behaviors and social capital was gathered through interviews, which are prone to recall bias and social desirability bias. Multivariate analyses were conducted to account for various demographic, clinical, and behavioral confounders; however, residual confounding from unmeasured variables, including the severity of heart failure symptoms (New York Heart Association Functional Class), cognitive function, mental health status, or caregiver involvement, may persist. Furthermore, despite implementing a stratified multistage sampling framework and randomization in the early stages, the last stage utilized quota sampling, potentially introducing selection bias. Lastly, the study lacks objective clinical outcomes, including hospitalization rates, mortality rates, or biomarker data, which limits the evaluation of the clinical outcomes associated with higher social capital.

## 5 Conclusions

The majority of heart failure patients suffered from inadequate self-care across all of its domains, especially those living in rural areas, those with a shorter duration of cardiologist surveillance, and those with a higher number of comorbidities. Community participation and proactive engagement in society contributed to all domains of self-care behaviors. The strength of connections with neighbors influenced maintenance and symptom management, while connections with coworkers impacted maintenance and symptom perception. Feeling of safety and trust in society impacted both maintenance behaviors and symptom perception. Additionally, connections with family and friends influenced maintenance behaviors. Higher scores in total social capital, proactive engagement, social trust and safety, along with stronger connections with neighbors and coworkers, were positively correlated with increased self-care confidence among heart failure patients.

## Funding

No funding was received for conducting this study.

## Ethics approval

The Research Ethics Committees of the School of Medicine–Shiraz University of Medical Sciences approved the present study with the following number: IR.SUMS.MED.REC.1404.205. The research was conducted in accordance with the ethical principles outlined in the Declaration of Helsinki of 1964 and its subsequent amendments, or comparable ethical standards.

## Data and/or code availability

Analyzed data that support the findings of this study can be made available upon request from the corresponding author.

## Prior presentation

None.

## Consent to participate

Informed consent was obtained from all individual participants included in the study.

## CRediT authorship contribution statement

**Amirhossein Saem:** Writing – original draft, Validation, Software, Methodology, Formal analysis, Data curation, Conceptualization. **Hamed Bazrafshan Drissi:** Writing – review & editing, Validation, Supervision, Resources, Project administration. **Armin Sharifi:** Writing – original draft, Investigation, Data curation. **Javad Kojuri:** Writing – review & editing, Validation, Resources. **Alireza Salehi:** Writing – review & editing, Supervision, Project administration, Methodology, Conceptualization.

## Declaration of competing interest

The authors declare that they have no known competing financial interests or personal relationships that could have appeared to influence the work reported in this paper.

## Appendix Supplementary materials

Image, application 1
