# Supplementary material for: The association between social capital and self-care behaviors in patients with heart failure: A cross-sectional study
Source: Int J Nurs Stud Adv. 2025 Dec 21;10:100478. doi: 10.1016/j.ijnsa.2025.100478 (PMC12997190; doi:10.1016/j.ijnsa.2025.100478)
Supplement: Supplementary file 1 [file mmc1.docx]

**Supplementary Material to “The Significance of Social Capital in the Self-care of Heart Failure Patients: Dive into the bottom of social relations, communications, and interactions”**


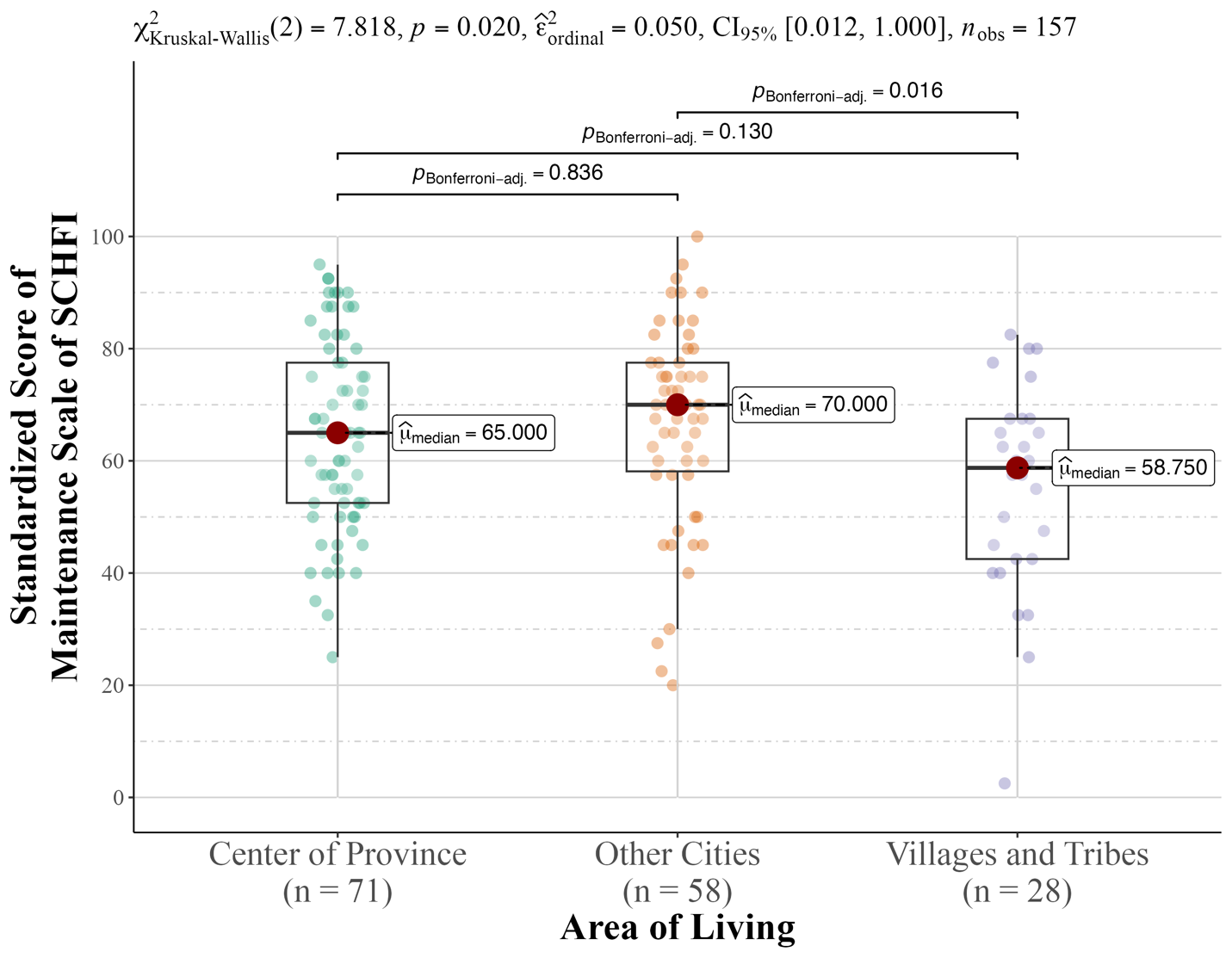


**Figure S1. Comparison of Self-care Maintenance Scores among Heart Failure Patients Living in Different Geographic Areas.** Heart failure patients who live in Villages and Tribes have lower self-care maintenance scores, with a median of 58.75 (IQR: 42.50 – 67.50). In comparison, those living in cities have a median score of 70 (IQR: 58.12 – 77.50), while patients in provincial centers have a median score of 65 (IQR: 52.50 – 77.50). This data shows the differences in self-care maintenance based on where patients live.


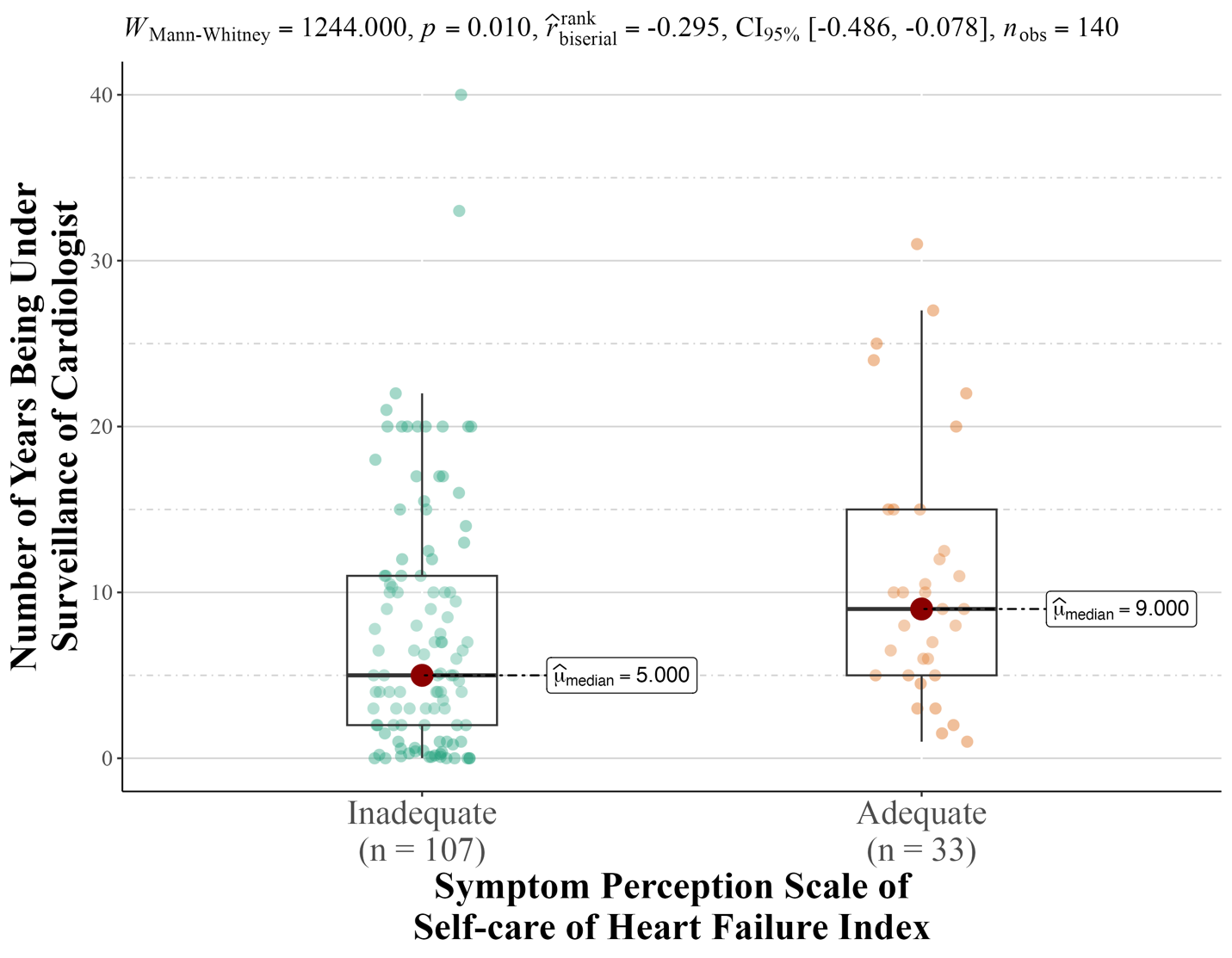


**Figure S2. Comparing Duration of Heart Failure Surveillance by Cardiologists among Patients with Adequate and Inadequate Symptom Perception.** Patients with adequate symptom perception had a median of 9 years (IQR: 5 – 15) of cardiology surveillance compared to 5 years (IQR: 2 – 11) in those with inadequate symptom perception, with a p-value of 0.010.

**Table S1.** **Subgroup Analysis of Social Capital and Self-Care of Heart Failure Measurements Among Hospitalized and Non-hospitalized Patients**

| **Validated Instruments** | | **Hospitalized Patients** | **Non-hospitalized Patients** |  |
| --- | --- | --- | --- | --- |
|  |  |  |  | **p-value**^1^ |
| **Total Score of Social Capital**, mean ± SD | | 41.58 ± 19.31 | 47.65 ± 14.71 | 0.143^*^ |
| **Social Capital Domains** | |  | | |
|  | *Community Participation*, median [IQR] | 32.26 [28.49–55.27] | 47.22 [38.89–58.07] | 0.135 |
|  | *Pro-activity in Social Contexts*, mean ± SD | 49.21 ± 24.64 | 52.23 ± 19.18 | 0.574^*^ |
|  | *Feelings of Trust and Safety*, median [IQR] | 53.33 [33.33–60] | 60.00 [46.67–80] | **0.008^$^** |
|  | *Neighborhood Connections*, median [IQR] | 33.33 [13.33–43.33] | 46.67 [26.67–60] | 0.068 |
|  | *Family and Friends Connections*, median [IQR] | 22.22 [22.22–55.56] | 44.44 [33.33–66.67] | 0.152 |
|  | *Tolerance of Diversity*, median [IQR] | 50 [25–66.67] | 50 [33.33–66.67] | 0.961 |
|  | *Value of Life*, median [IQR] | 50 [33.33–66.67] | 50 [33.33–66.67] | 0.504 |
|  | *Work Connections*, median [IQR] | 77.78 [55.56–88.89] | 66.67 [44.44–77.78] | 0.383 |
| **Self-care Domains** | |  | | |
|  | Maintenance Scale, median [IQR] | 60 [40–75] | 65 [52.5–76.88] | 0.277 |
|  | Symptom Perception, mean ± SD | 56.33 ± 22.68 | 55.7 ± 17.91 | 0.903^*^ |
|  | Symptom Management, mean ± SD | 64.96 ± 21.93 | 57.61 ± 17.61 | 0.151^*^ |
| **Self-care Confidence**, median [IQR] | | 61.25 [44.38–91.88] | 68.75 [50–83.12] | 0.737 |

^1^ All p-values less than 0.05 are considered statistically significant.

^*^ *P-value* of the independent two-sample t-test, comparing mean differences between hospital and clinic subgroups.

^$^ *P-value* of the Kruskal-Wallis test, a non-parametric test, compares rank differences between hospital and clinic subgroups.

*Abbreviations. SE: standard error, COPD: chronic obstructive pulmonary disease, HFrEF: heart failure with reduced ejection fraction, HFpEF: heart failure with preserved ejection fraction, HFmrEF: Heart Failure with mildly reduced ejection fraction.*

**

Figure S3. Associations between additional social capital subscales and self-care domains in patients with heart failure. (A)** Differences in *Total scores of social capital* between patients with adequate versus inadequate self-care scores across three domains of the Self-Care of Heart Failure index: self-care maintenance, symptom perception, and management, as well as self-care confidence. **(B)** Differences in *Tolerance of Diversity* subscale scores of social capital between patients with adequate versus inadequate self-care scores in each self-care domain. **(C)** Differences in *Perceived* *Value of Life* subscale scores of social capital between patients with adequate versus inadequate scores in each self-care domain. Box plots display medians, interquartile ranges, and outliers; *p*-values are from group comparisons.
